# Igneous: Distributed dense 3D segmentation meshing, neuron skeletonization, and hierarchical downsampling

**DOI:** 10.3389/fncir.2022.977700

**Published:** 2022-11-25

**Authors:** William Silversmith, Aleksandar Zlateski, J. Alexander Bae, Ignacio Tartavull, Nico Kemnitz, Jingpeng Wu, H. Sebastian Seung

**Affiliations:** ^1^Princeton Neuroscience Institute, Princeton University, Princeton, NJ, United States; ^2^Department of Electrical Engineering and Computer Science, Massachusetts Institute of Technology, Cambridge, MA, United States; ^3^Department of Electrical and Computer Engineering, Princeton University, Princeton, NJ, United States; ^4^Department of Computer Science, Princeton University, Princeton, NJ, United States

**Keywords:** meshing, skeletonization, neuroscience, connectomics, image processing, cloud computing, distributed computing, compression

## Abstract

Three-dimensional electron microscopy images of brain tissue and their dense segmentations are now petascale and growing. These volumes require the mass production of dense segmentation-derived neuron skeletons, multi-resolution meshes, image hierarchies (for both modalities) for visualization and analysis, and tools to manage the large amount of data. However, open tools for large-scale meshing, skeletonization, and data management have been missing. Igneous is a Python-based distributed computing framework that enables economical meshing, skeletonization, image hierarchy creation, and data management using cloud or cluster computing that has been proven to scale horizontally. We sketch Igneous's computing framework, show how to use it, and characterize its performance and data storage.

## 1. Introduction

Over the past decade, advances in the dense reconstruction of microscale neural circuits, a field known as connectomics, have produced increasingly large stacks of electron microscopy images derived from thinly sliced plasticized brain tissue (Pfister et al., 2014). In recent years, several large datasets have appeared, including the whole brain (Zheng et al., 2018) and hemibrain (Xu et al., 2020) versions of *Drosophila melanogaster*. In 2021, a cubic millimeter dataset of mouse primary visual cortex (MICrONS Consortium et al., 2021) and a petascale fragment of human cerebral cortex (Shapson-Coe et al., 2021) were made available as pre-prints, the largest datasets to date. There are even ongoing discussions on imaging a whole mouse brain, a volume hundreds of times larger than either of those (Abbott et al., 2020; Rose Li and Associates Inc., 2021).

Such volumes are far larger than the capacity of existing single machines and so require the use of cloud storage or other large networked filesystems. Not only is hardware required to store these massive datasets, but also software to visualize, process, and manage them. Efficiently producing and managing datasets of this size is a key challenge for the connectomics field as it scales to acquire the complete wiring diagram of higher order organisms.

There are many types of data involved in such investigations (Pfister et al., 2014; Beyer et al., 2022), but at the microscale, they typically consist of stacks of single-channel electron or multi-channel confocal microscopy images. Electron micrograph stacks are processed into (usually dense) per-voxel 32 or 64-bit labelings (“segmentations”) and intermediate representations such as three-channel 32-bit floating point voxel affinities. From the segmentation are derived surface meshes for 3D visualization and skeletons (Tagliasacchi et al., 2016) (stick figure centerline representations of geometries) which have analytical, visualization, and graphical user interface uses. Volumes may be annotated with points and lines (such as for describing synapses or other sub-cellular compartments).

Many systems have been published that excel at handling large-scale image data. To our knowledge, there are no publicly available systems capable of large-scale dense segmentation meshing and skeletonization (see Related Work). Our innovation is the release of fully functional, open source, and easy-to-use software that mass produces meshes and skeletons directly from segmentation images and independently of each other. It is not necessary to produce a set of meshes before producing skeletons nor vice versa. These two innovations are embedded within a larger system that produces and manages Neuroglancer viewable volumes (Maitin-Shepard et al., 2021).

**Figure 1:**
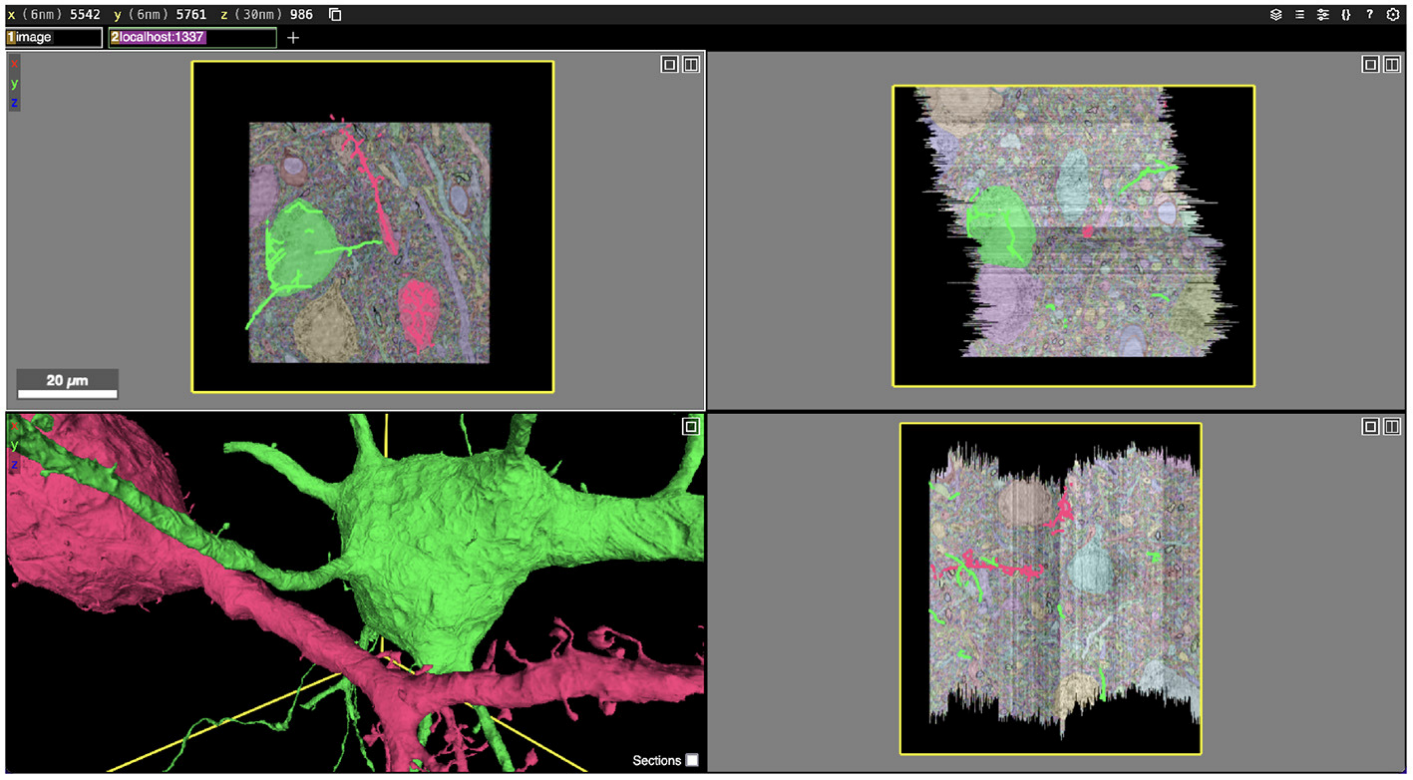
Screenshot of an Igneous generated dataset viewed in Neuroglancer. Igneous is designed to produce complete Neuroglancer viewable datasets from images and segmentations. Above, two cells are selected from an unproofread automatic segmentation of the S1 dataset and displayed in Neuroglancer. Three zoomed out cross-sectional views (XY, XZ, and YZ planes going clockwise) are shown with overlaid electron microscope images and segmentation labels. Image pyramids, meshes, and skeletons (in 2D) can be seen in the bottom left panel.

Neuroglancer is a viewer that has been gaining popularity (see Figure 1). It is a lightweight static web page that is pointed at storage backends such as local web servers, Google Cloud Storage, and Amazon S3 (or S3 emulators) to pull in data. Neuroglancer's native format, Precomputed, is designed to make the client-side calculation of the necessary files' locations trivial without scanning a filesystem or querying a database. Precomputed divides the image into a regular grid of chunk files. It may also store groups of those files in a container “sharded” format that retains the random read access property (but not random write access) to reduce the load on the filesystem. A resolution hierarchy is also defined so that the entire image can be visualized at low resolution and refined as the viewer is zoomed in. Neuroglancer also provides specifications for visualizing 3D meshes, skeletons, and annotations with multi-resolution formats defined for meshes and annotations. However useful Neuroglancer is, it nonetheless doesn't come with a way to create, manage, or programmatically read the Precomputed format.

**Figure 2:**
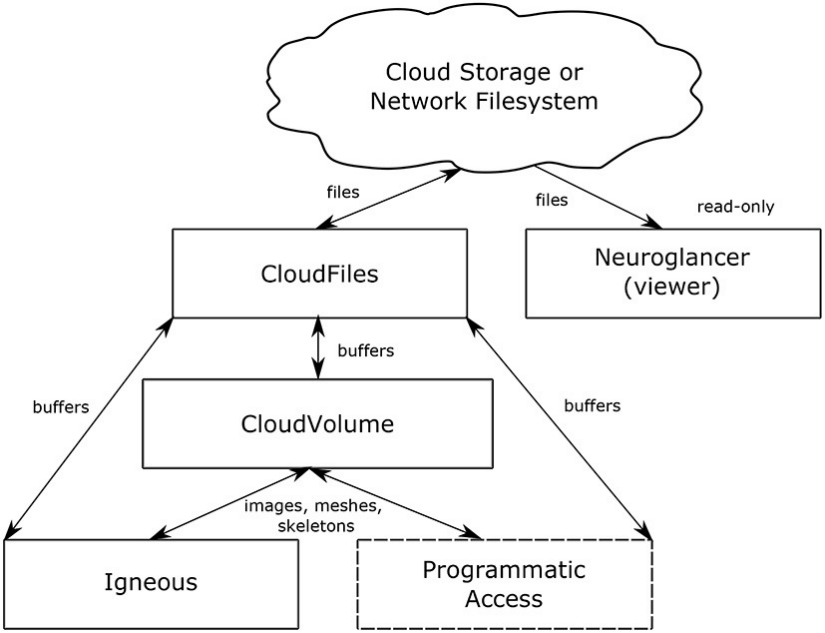
Data flow. The relationship between Igneous, CloudVolume, CloudFiles, and Neuroglancer. Arrows indicate the direction of data flow with reading flowing from top to bottom and writes flowing bottom to top. Neuroglancer is a separate application that only reads data. Igneous uses CloudVolume for high level primitives such as images, meshes, and skeletons and uses CloudFiles for low level file IO. The box “Programmatic Access” serves to indicate that CloudVolume and CloudFiles also provide programmatic access to the dataset for many other situations outside of Igneous.

We report Igneous^1^, an open-source software Python program that provides a critically needed scalable, low-cost, and easy-to-use computational pipeline for generating and managing bulk Precomputed data such as image pyramids, meshes, and skeletons. Much like its sister software chunkflow (Wu et al., 2021), which is used for generating segmentations, Igneous is robust to task failure and can be used with cheap unreliable cloud instances (sometimes called “preemptible” or “spot” instances). Igneous can be run completely locally or massively scaled in the cloud. It requires minimal setup and no maintenance between runs. As seen in Figure 2, it is completely independent of Neuroglancer itself, though it produces datasets that comply with the Neuroglancer Precomputed specifications.^2^

In this article, we will sketch Igneous' computational framework, show how to use it, describe our innovations in meshing and skeletonization of dense segmentation, and characterize the system's performance.

### 1.1. Related work

Many labs have developed separate solutions for storing, visualizing, and annotating datasets of ever-increasing size as contemporary commercial solutions were not adequately scalable, were missing features, or both. These tools operate on many different principles, and most of them have a method for importing images. However, there is limited support for meshing and skeletonization of dense segmentation in bulk as we will describe below.

To give a brief sketch of the landscape, these systems can be broadly characterized by the maximum data size they support (in-memory, disk, network filesystem / cloud storage), method of neural representation (skeletons, per-voxel image segmentation, network representation), their methods of visualization [single resolution image, image pyramid (Pietzsch et al., 2015; Sofroniew et al., 2022), precomputed or dynamic surface meshes, skeletons, volume rendering (Peng et al., 2010; Maitin-Shepard et al., 2021), or other], method of data storage [single file, chunks, random-access consolidated files (“shards”), versioning, and serverlessness], method of proofreading [skeleton tracing (Saalfeld et al., 2009; Boergens et al., 2017) and segment merging and splitting (Kim et al., 2014; Katz and Plaza, 2019; Dorkenwald et al., 2021), with variations within each category], and openness of access to reconstructions and proofreading prior to publication (public, semi-public, or internal only), and the different overlapping communities of proofreaders, viewers, and software developers centered around each tool. Proofreading systems for editing the annotations and segmentation may have additional storage requirements such as a graph of connected segments (Anderson et al., 2011; Ai-Awami et al., 2016; Dorkenwald et al., 2021). An excellent overview of the visualization landscape can be found in a recent survey by Beyer et al. (2022) and in the VAST paper by Berger et al. (2018).

To place Igneous in relation to these categories, it produces Neuroglancer Precomputed volumes that are stored in cloud or network storage. Neurons are represented by per-voxel annotations, precomputed multi-resolution surface meshes, and precomputed skeletons. The image data are stored as versionless chunks or shards (see Consolidating Files). Igneous is free software that can be used by anyone. See the Supplementary material for additional information.

#### 1.1.1. Meshing

Meshing is frequently available at the single machine level for many tools. However, there are few publicly available tools that provide meshing at scale. This may be because most available implementations of Marching Cubes (Lorensen and Cline, 1987) are applicable only to binary images or continuously valued images, which requires potentially thousands of iterative evaluations for a cutout of densely labeled segmentation. Despite the relative efficiency of Marching Cubes and similar algorithms, this repeated application to a cutout can result in long processing times as large amounts of redundant computation are incurred to produce a separate mesh for every label.

Additionally, once a mesh is produced it is too detailed. Marching Cubes produces one or more triangle faces for every foreground voxel in a volume. This poses problems for both the storage and display of objects as the mesh is supposed to be a lighter-weight representation than the dense labeling. With three floats per vertex and three integers per triangle face compared with a single integer per voxel, an unrefined mesh may be larger than the object's image representation. A natural solution to this problem is mesh simplification, but many such algorithms are prone to change mesh topology and existing implementations are often hard to use for a variety of reasons including the availability of language bindings and performance.

However, these problems are not intractable. Several large scale multi-resolution meshings have been published as pre-prints by the Google Connectomics team (Xu et al., 2020; MICrONS Consortium et al., 2021; Shapson-Coe et al., 2021). At least one sparse segmentation published using WebKnossos (Boergens et al., 2017) has precomputed meshes (Helmstaedter et al., 2013) (larger densely labeled volumes appear to rely on on-demand meshing). However, the meshing engines that created these datasets are not publicly available.

A distributed meshing tool called mesh-deco^3^ by Matelsky works by passing extracted binary images to mesh workers. This approach is compatible with sparse meshing, but will be inefficient for dense meshing.

Other tools have found workarounds for the difficulties of bulk meshing. Some proofreading tools (e.g., CATMAID, Saalfeld et al., 2009) rely on skeleton tracing and therefore have a less pressing need for bulk meshing. NeuTu found a creative method for rapidly visualizing segments by rendering all surface points of an object as a set of spheres in cases where meshes are not available (Zhao et al., 2018).

A few projects have built Neuroglancer multi-resolution mesh generation capabilities that used pre-existing base meshes. Sidky's neurogen^4^ and Ackerman's Multi-Resolution-Mesh-Creator^5^ for DVID (Katz and Plaza, 2019) demonstrated the viability of using a mesh simplification strategy. Jagannathan's pyroglancer^6^ also has a multi-resolution mesh creation capability.

Our sister project chunkflow (Wu et al., 2021), which uses the same meshing library that we use, also has the ability to densely mesh, but at single resolution without shards.

Thus, it could be said that the access and ability to perform large-scale meshing of dense segmentation has been very uneven even though in principle versions of the algorithm and open source software have been available. We report a method for efficiently and economically mass producing large, multi-resolution simplified meshes from dense segmentation that is publicly available and easy to install.

#### 1.1.2. Skeletonization

Extracting skeletons from segmented neurons have obvious benefits for neuroanatomical analysis as they are simpler to manipulate and represent the structural connectivity of the interior of a neuron rather than of the surface. However, computing them in quantity has been a challenge for the field. The most popular skeletonization algorithm in connectomics studies is TEASAR (Sato et al., 2000; Bitter et al., 2001; Zhao and Plaza, 2014). However, implementations of TEASAR are often memory hungry and slow. As with meshing, this is partly due to existing implementations which only accept binary images and thus need to iteratively evaluate a densely labeled volume. However, there are other elements of the algorithm that present problems, such as requiring the construction of large graphs of connected voxels and then performing operations on this graph. Thus, usually only a select fraction of objects in a segmentation can be practically skeletonized, and that volume is usually of limited size or resolution.

Some existing C++ implementations of TEASAR can be found in NeuTu^7^ and Skeletopyze.^8^ A Python implementation by Bae is found in Skeletonization.^9^ A particularly interesting Julia implementation by Wu et al. (2022) uses bit packing and sparse graph representations to enable the sparse skeletonization of large neurons with full context by making it practical to fit the whole neuron in memory.

In the past few years, some alternative approaches which extract skeletons from meshes have appeared in tooling. The approach by Dorkenwald et al. in MeshParty (Dorkenwald et al., 2020) could be described as “Mesh TEASAR” as it uses the surface mesh triangle graph instead of a voxel connectivity graph. Skeletor by Schlegel and Kazimiers (2021) contains several published algorithms for extracting a skeleton from a mesh (including a “Mesh TEASAR” implementation). These approaches are promising and warrant further exploration.

A popular voxel thinning algorithm implementation, Skeletonize3d^10^ by Ignacio Arganda-Carrera can be found in Fiji based on the algorithm by Lee and Kashyap (1994) and an ITK implementation by Hanno Homann.^11^ Another innovative technique by Matejek et al. called SynapseAware (Matejek et al., 2019) modifies a voxel thinning algorithm to preserve pathways between synapses specifically to optimize the Neural Reconstruction Integrity (NRI) metric for the resultant skeletons (Reilly et al., 2018).

We report a method of mass producing high-quality skeletons directly from dense segmentation images by using a fast memory optimized TEASAR implementation and a novel chunking and stitching strategy. To our knowledge, no other publicly available tool has demonstrated this capability.

#### 1.1.3. Images

Image handling is so basic to connectomics and other kinds of investigations that there is a very large amount of prior work both within and outside of the field. Therefore, we will only briefly treat the most closely related systems to confine ourselves to the available space. Again, please consult Berger et al. (2018) and Beyer et al. (2022) for more information.

Nearly all major connectomics systems support the display of image pyramids. Neuroglancer, CATMAID (Saalfeld et al., 2009), NeuTu/DVID (Zhao et al., 2018; Katz and Plaza, 2019), Knossos (Helmstaedter et al., 2011), WebKnossos (Boergens et al., 2017), PyKnossos (Wanner et al., 2016), BigDataViewer (Pietzsch et al., 2015), Vaa3d (Peng et al., 2010), BossDB (Hider et al., 2019), Omni (Shearer, 2009), VAST (Berger et al., 2018), RECONSTRUCT (Fiala, 2005), VikingViewer (Anderson et al., 2011), Dojo (Haehn et al., 2014), Ilastik (Berg et al., 2019), IMOD (Kremer et al., 1996), TrakEM2 (Cardona et al., 2012), ITK-SNAP (Yushkevich et al., 2006), and SSECRETT/NeuroTrace (Jeong et al., 2010) all support the storage and display of image pyramids of electron micrographs. Most of these also support the display of segmentation overlays as well. Usually, each level of the pyramid is chunked so that subsets of an image can be retrieved efficiently.

Eyewire (Kim et al., 2014) does not support the display of the entire image at once and displays only small blocks at a time. It relies on a dynamically updated overview mesh for providing overall context for each cell. Omni is used in conjunction with tasks that require large image context.

Many of these tools use a custom file format for storing the image pyramid. In some cases, the image viewers are compatible with multiple formats. For example, Neuroglancer is currently compatible with the following imaging formats^12^: BossDB, BrainMaps (Google's internal format), DVID, N5^13^, nifti^14^, Precomputed (Neuroglancer's native format), Render^15^, and zarr (Miles et al., 2022).

Several tools provide guidance or a tool for aiding in the import and processing of a new image dataset. CATMAID provides an importer tool^16^, DVID has a built-in downsampler^17^ and can be run in clustered fashion. Ingest clients are provided by BossDB (ingest-client^18^) and WebKnossos (wkCuber^19^).

We report a tool for downsampling and managing cloud storage hosted Precomputed format images and segmentations that is proven to scale to hundreds of teravoxels and supports sharded images (see Condensing Files). To our knowledge, Igneous (*via* CloudVolume) is the first publicly available tool to support the Compresso (Matejek et al., 2017) dense segmentation compression codec.

## 2. Methods

**Figure 3:**
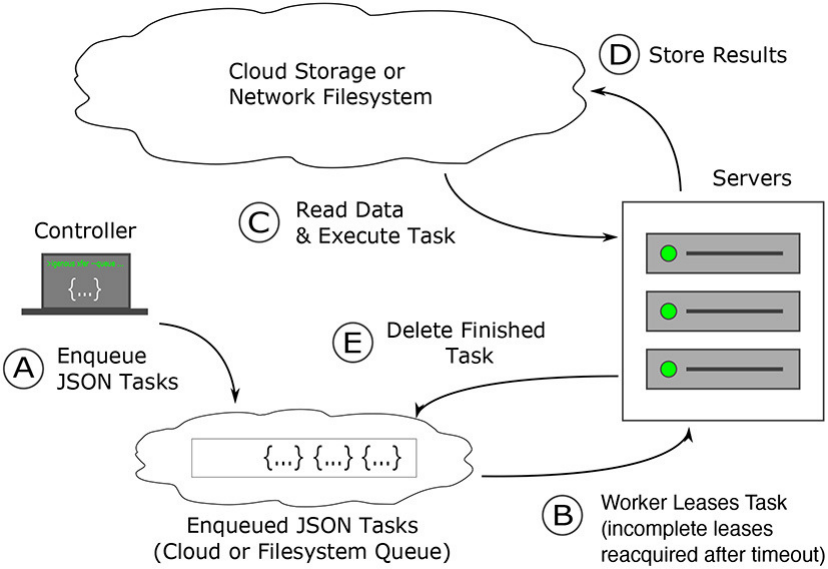
How Igneous works: task creation and distribution. Task distribution and execution are robust to failure. **(A)** Dependency free tasks are generated as JSON from a lightweight machine and inserted into a cloud queue (Amazon Web Services' SQS) or into a the local file system, which operates similarly **(B)** workers from a pre-configured cluster (usually controlled *via* Kubernetes or SLURM) continuously attempt to acquire a time-based lease on tasks. Once the lease expires, the task is again available to be leased. **(C)** Once a task lease is acquired, the worker uses the instructions in the task to fetch data from cloud storage and executes the task specified procedure against it **(D)** on finishing execution, the results are written back into the cloud. **(E)** The task is then deleted from the cloud queue to prevent redundant re-execution. When all tasks are deleted the job is complete.

Igneous uses a dependency-free task queue in order to assign and distribute work. A schematic of how this works can be seen in Figure 3. Dependency freedom is possible because the production and management of image pyramids, meshes, and skeletons can be broken down into either spatially chunked tasks without significant overlap or into non-overlapping ranges of integer labels which enables efficient parallelization. Some operations, such as the production of a small image pyramid can be performed in a single pass. More complex operations, such as building larger image pyramids, meshing, or skeletonization, build on top of previous results either directly or in map and reduce passes (termed “forging” and “merging,” respectively, within Igneous).

Igneous's queue, python-task-queue^20^, converts annotated python functions or objects into a lightweight JSON stream that can be submitted to a cloud queue (such as Amazon SQS) or to our on-disk emulation of SQS we term FileQueue. These queues provide a guarantee of durability; a task will either be executed or recycled upon failure. If a task fails, eventually the time-based lease will expire and another worker will re-execute the task. This possibility of re-execution requires tasks to be idempotent. Dependency-free operation allows for the parallelization of task generation, insertion, and execution, which becomes convenient for very large datasets that may require hundreds of thousands or millions of tasks.

Igneous is highly versatile and easily operates in different environments. For small jobs, usually running it locally from the command line is sufficient. For larger jobs, it has been successfully used with SLURM (Yoo et al., 2003) in Princeton's Della cluster^21^ and Docker^22^/Kubernetes^23^ using Google Cloud Platform.^24^ As the filesystem and SQS are always available and reasonably durable, jobs can be stopped and resumed at will.

So long as workers can access the queue and datastore, they can be located anywhere in the world. This enables hybrid computing using local resources, university clusters, and cloud platforms simultaneously. FileQueue enables Igneous to also be used in limited environments as it only requires a filesystem that provides POSIX advisory file locking. For example, national supercomputing centers may restrict internet connectivity on worker nodes and may not allow the installation of a persistent queuing service, but nonetheless provide a common filesystem.

Igneous can theoretically work with any Key-Value store. It uses CloudVolume (Silversmith et al., 2021b) and CloudFiles^25^ software which provide threaded IO to the filesystem, Google Cloud Storage, Amazon S3, S3 emulators, and static HTTP file servers. Thus, data can be stored and shuffled to the most convenient or cost-efficient location whether that's local, in the cloud, or at an on-premises network file system or S3 emulator.

The relationship between Neuroglancer, Igneous, CloudVolume, and CloudFiles can be seen in Figure 2. Igneous uses CloudVolume and CloudFiles to read and write data to cloud storage or a network filesystem in order to create Neuroglancer readable volumes. CloudVolume, used to manage higher order primitives like image cutouts, meshes, and skeletons, uses CloudFiles for file IO. Neuroglancer, a viewer for 3D datasets, is a web page that can independently read Igenous generated datasets directly from cloud storage or from an HTTP server. Neuroglancer is a completely separate code base from Igenous, CloudVolume, and CloudFiles. The “Programmatic Access” element indicates that CloudVolume and CloudFiles provide programmatic access to the dataset outside of their use in Igneous (beyond the scope of this article).

### 2.1. Condensing files (“sharding”)

Random read access to large datasets is often achieved by chunking large images into many smaller files or writing out each mesh, skeleton, or other derivatives of a label as one or more files. This results in problems for the filesystem when the number of files becomes large. Even with cloud storage, as of this writing, file creation costs between $5^26^ and $6.50^27^ per a million files on several cloud providers on active storage (usually the default tier). When hundreds of millions or billions of files are created, the initial upload costs can be more than the cost of the computation.

While it's difficult to impute the financial cost directly to the stress put on the storage system, it is notable that object storage systems often use an erasure coding (Balaji et al., 2018) or replication scheme that creates multiple copies or parity fragments to be able to reconstruct each file in the event of bit flips or damage to machines and hard disks. Frequently, the metadata used to locate distributed copies or file fragments are themselves replicated several times. Thus, there is a constant storage multiplier attached to each file uploaded. Sometimes, even if a large dataset can be created, deleting it can be difficult on some systems, incurring delays or additional costs.

**Figure 4:**
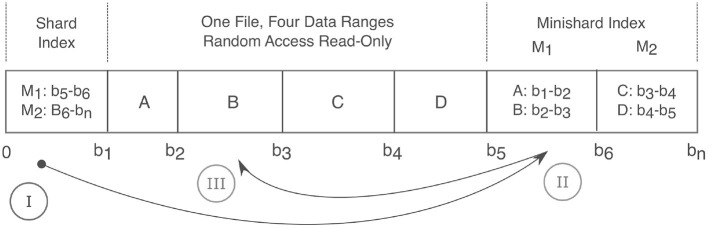
Anatomy of a Shard. A shard condenses many files into a single read-only random access file and thereby relieves strain on filesystems. Integer labels are mapped to a given shard filename *via* a hash function. Random read access is achieved *via* a two level index that maps an integer label to a corresponding byte range. The index consists of the first fixed size “Shard Index” that then maps to variable size “Minishard Indices” which contain the label to byte range mappings. In the drawing above, A,B,C,D are integers and are positioned over their corresponding byte ranges. At the bottom, 0 to *b*_*n*_ connote the byte offset with *b*_*n*_ being the end of the file. The roman numerals show the sequence of accesses. Prior to caching, three requests are needed to fetch label B in byte range *b*_2_ to *b*_3_. (I) The shard index is accessed, which points to the (II) *M*_1_ minishard, which (III) locates label B between bytes *b*_2_ and *b*_3_.

As a mitigation, Neuroglancer adopted an approach that condenses many individual files into random-read (but not random-write) files called “shards”^28^ (see Figure 4). Shards reduce the number of files in a dataset by several orders of magnitude. This kind of approach is becoming more popular and implementations are being discussed in prominent projects like Zarr.^29^ Techniques similar to sharding can be implemented by a filesystem to cope with large numbers of small files, so shards could be viewed as a client-side emulation of a filesystem feature.

Igneous provides methods for creating shard files for each data type it supports (images, meshes, and skeletons) and CloudVolume is capable of reading them. As producing shards is more complicated and removes random access writes, it is usually hidden behind a flag (e.g., --sharded) or a separate command (e.g., merge-sharded).

### 2.2. Supported data encodings

Igneous supports all encoding methods that are currently supported in Neuroglancer Precomputed.

#### 2.2.1. Microscopy images

Electron microscopy and other natural images are often represented as single-channel (grayscale) 8-bit or 16-bit unsigned integers. Igneous supports compressing chunk files using raw+gzip, raw+brotli (Alakuijala and Szabadka, 2016), png^30^, and jpeg^31^ (where raw means a serialized array).

We currently use the SIMD^32^ accelerated deflate^33^ library (based on libdeflate^34^) to accelerate gzip, pyspng-seunglab^35^ (based on libspng^36^ and pyspng^37^) to accelerate PNG, and SIMD accelerated simplejpeg^38^ (based on libjpeg-turbo^39^) to accelerate JPEG codec performance.

#### 2.2.2. Segmentation images

Connectomics segmentation images have uncommon image statistics in that they are often 64-bit unsigned integers, densely labeled so that nearly every voxel is foreground and contain potentially billions of labels that are densely packed smoothly varying organic shapes. This creates a densely packed image with simple statistics where most adjacent voxels are equal which results in an amusing contradiction. The large data width of the segmentation means that it is eight times larger than the image it was derived from but it compresses so excellently, that it is much smaller on disk.

Igneous and Neuroglancer support three compression schemes that can be layered with gzip or brotli compression for segmentation images: raw, compressed_segmentation^40^, and compresso. The latter two codecs were designed specifically with connectomics segmentations in mind.

compressed_segmentation renumbers and bit packs small 3D regions within the image (often 8 × 8 × 8 voxels) to vastly reduce the size of the representation. It works extremely well with second-stage compression which often results in both smaller data and faster IO overall. It is also randomly accessible, and so is used by Neuroglancer in order to pack more image regions into the GPU. We use a Cython wrapper (Behnel et al., 2011) around Jeremy Maitin-Shepard's C++ encoder and Stephen Plaza's C++ decoder for our codec.^41^

Compresso (Matejek et al., 2017) was designed explicitly for two-stage high compression of connectomics segmentation. Compresso represents the boundary between segments as a boolean bit packed field. Long runs of zeros are run-length encoded. The 4-connected components within that field are mapped to a corresponding label. Boundary voxels are decoded with reference to their neighbors, or if the location is ambiguous, by storing their label. We adapted the Compresso algorithm and modified pre-existing code to create a Cython/C++ codec.^42^.

We've found that high-resolution images (mips 0 and 1) typically compress better with compresso than with compressed_segmentation. Lower resolution images, since they become noisier, do not compress as well and eventually compressed_segmentation achieves higher compression. A hybrid strategy of using compresso for high-resolution layers and compressed_segmentation for lower resolution layers could be used to achieve higher compression overall.

#### 2.2.3. Meshes

Meshes generated by Igneous are written in the triangle soup Precomputed format^43^. They can be compressed with gzip or brotli for single-resolution meshes, and with Draco^44^ for multi-resolution meshes. It is possible to convert meshes to the popular Wavefront OBJ or PLY formats on demand using CloudVolume.

#### 2.2.4. Skeletons

Skeletons are written in the Precomputed format^45^ and can be compressed with gzip or brotli. They are stored as an undirected vertex graph with vertex attributes. They can be converted to the widely used tree-based SWC format on demand using CloudVolume (loops may cause errors).

### 2.3. Downsampling

Large volumetric images do not fit in RAM and are not practical to download for visualization. It is also desirable to perform some types of processing on lower resolution images. Thus, it is necessary to create an image pyramid of lower resolution images that can be downloaded selectively (e.g., depending on the viewer's current zoom level). Typically, each successive resolution level (or “mip”^46^) is downsampled using 2 × 2 (2D) or 2 × 2 × 2 (3D) voxel pooling. For Neuroglancer, mip 0 is the highest resolution layer, with higher integers indicating successively lower resolution layers.

In calculating these mip levels, we use average pooling for natural (electron microscopy) images and recursive mode pooling for labeled (segmentation) images. Uncompressed, a pyramid of 2 × 2 downsamples requires 33% additional storage over the base image. A pyramid of 2 × 2 × 2 downsamples will require 14% more. In both cases, mip 1 is the dominant contributor to the additional storage (25% for 2 × 2, 12.5% for 2 × 2 × 2).

Multiple mip levels can be generated from a single task if the task is large enough. This is advantageous because (a) it takes fewer runs to generate all mip levels (b) fewer tasks need to be generated and managed (c) the average pooling algorithm can avoid integer truncation. Nonetheless, it's often not possible to generate all mip levels in a single task as each additional level exponentially multiplies the required memory by 4–8 times. Therefore, by default, five levels are generated and another set can be generated on top of them in a process we term “superdownsampling.”

Integer truncation becomes significant in very large volumes that may have 10+ mip levels. If it is not accounted for, the lowest resolution levels are noticeably darker than the base level as up to 0.75 luminance units can be lost per voxel at each level in 2 × 2 pooling or 0.875 units in 2 × 2 × 2 pooling. At 10 mips, this amounts to a potential loss of 7.5 units in 2 × 2 and 8.75 for 2 × 2 × 2. Our average pooling implementation in the tinybrain^47^ library mitigates this issue by accumulating sums for each mip level before dividing.

2 × 2 × 2 downsampling can lead to ghosting around the edges of a slice as extensions of one slice regions can be averaged with several zeros from a gap in an adjacent slice. We offer a sparse downsampling mode that skips counting zero-valued voxels in the average.

For sharded volumes, due to the large memory requirement for holding a single shard in memory, Igneous can only generate a single mip level at a time. Generating additional mip levels would require holding exponentially larger numbers of shards in memory for each mip level unless the shards shrink at each mip level, reducing their utility. This requirement means that producing a sharded mip incurs integer truncation errors. However, it is possible to produce unsharded mips from sharded mips and later condense them.

### 2.4. Meshing

While visualizing cross sections of 3D segmentation are informative, it is difficult to understand the overall shape of a neuron outside of a series of local contexts without a visual representation of the whole object. Meshing, creating a 3D surface representation consisting of vertices and faces from a segmentation, provides a light-weight method of visualizing neurons that may span an entire dataset as only the surface of the object of interest needs to be represented.

Neuroglancer offers three different formats for representing meshes: single-resolution unsharded, multi-resolution unsharded, and multi-resolution sharded (single-resolution sharded is not supported). The single resolution format allows for multiple mesh fragments for each segmentation label to be written as separate files and then pointed to by a manifest JSON file with a well-known filename (“SEGID:0”). The multi-resolution formats are different in that each mesh data file must contain the whole mesh and potentially additional lower resolution meshes addressable in an octree format. In the unsharded version, each label has a data file and an index file that gives instructions for reading the data file's octree. In the sharded format, a mesh's index file and data file are concatenated and grouped with many other labels's meshes. The benefit of multi-resolution files is that Neuroglancer can display lower resolution meshes when the viewer is zoomed out, which can allow for a higher performance display of large or numerous meshes.

We produce meshes by applying a specialized variant of the marching cubes algorithm (Lorensen and Cline, 1987) to segmentation images and then simplify the results. Two passes are needed. As it is impossible to produce a mesh spanning large datasets within the memory of a single machine, the image must be divided into smaller tasks and then merged. The first pass produces mesh fragments derived from small spatial areas and writes them to storage. The second pass aggregates the mesh fragments to create the final output (and in the case of multi-resolution meshes, also generates a hierarchy of simplified meshes).

Typically we begin with downsampling the segmentation image to use a near-isotropic mip level for meshing. While any mip level can be used, downsampling vastly reduces the amount of data to be processed, often by 64 × , while retaining a reasonable amount of detail. The selected mip level's image is then divided into a regular grid of tasks that each overlap on their positive axis face by one voxel.

We wrote the free software zmesh^48^ library to produce our meshes. zmesh is based on zi_lib^49^, which was first used in the Omni (Shearer, 2009) neural reconstruction proofreading software and then in Eyewire (Kim et al., 2014), an online crowdsourced proofreading platform. It implements a specialized version of the marching cubes algorithm (Lorensen and Cline, 1987) which efficiently handles multi-labeled images. This allows for all meshes to be generated in a single pass instead of thousands. The resultant mesh fragments are then simplified using the methods of Garland and Heckbert (1997) and Hoppe (1999) modified to retain topological integrity by filtering out unsound simplifications.

The one voxel overlap causes marching cubes to output mesh fragments that can be trivially stitched. Each fragment is then simplified before being serialized and compressed with gzip. Single resolution meshes are serialized in the legacy Precomputed format. Meshes written to the multi-resolution format are Draco compressed with integer position attributes using DracoPy.^50^ To generate a resolution hierarchy, we repeatedly apply pyfqmr^51^ to the base mesh with increasingly aggressive settings.

### 2.5. Skeletonization

Skeletons are stick-figure centerline representations of neurons. They are often represented as trees or graphs with vertices that are localized in space. They have many uses in analyzing the anatomy and function of neurons. For example, they can be used to compute basic properties such as cable length and wire diameter (when the skeleton is a medial axis transform). They can also be used to define functional sections of neurite during analysis such as dendrite and axon or to model electrical compartments. Skeletons can be used to guide cameras in 3D renderings and in proofreading neural circuits (as in CATMAID and WebKnossos).

However, mass-producing large skeletons from dense segmentation images is difficult due to both performance and memory requirements. Most image skeletonization algorithms require holding the entire image in memory, which is impossible at near petavoxel scale. The most popular skeletonization algorithm in the connectomics field is the TEASAR algorithm (Sato et al., 2000; Bitter et al., 2001), due to its flexibility in skeletonization different shapes with parameterization (Zhao and Plaza, 2014). However, the previously available implementations of TEASAR are very slow and require high memory usage. Furthermore, like meshes, mass production of skeletons can produce hundreds of millions of files, which results in difficulties with the filesystem or high cost on cloud storage. These problems previously made the mass production of skeletons impractical. However, we have been able to overcome these limitations and can now mass produce skeletons directly from segmentation images and without the need for a meshing step.

We developed Kimimaro (Silversmith et al., 2021a), a fast Python, Cython, and C++ based TEASAR-like algorithm that can process densely labeled segmentation images at hundreds of kilovoxels per a second with memory usage low enough to use on consumer hardware with a 512^3^ voxel cutout. However, even with improvements in the core algorithm, it was still not feasible to skeletonize the entire image in one shot due to memory constraints. Thus, we chunk the image into a regular grid of 512^3^ voxel tasks and stitch the results together in a second pass, similar to meshing. For this approach to work, several conditions must be satisfied (a) the stitching process must be made reliable (in the sense that the right connection between tasks is always made) and efficient (b) the tasks must be large enough to encompass significant morphological features or else skeletonization may go awry from lack of context (c) stitched skeletons should be free of loops.

**Figure 5:**
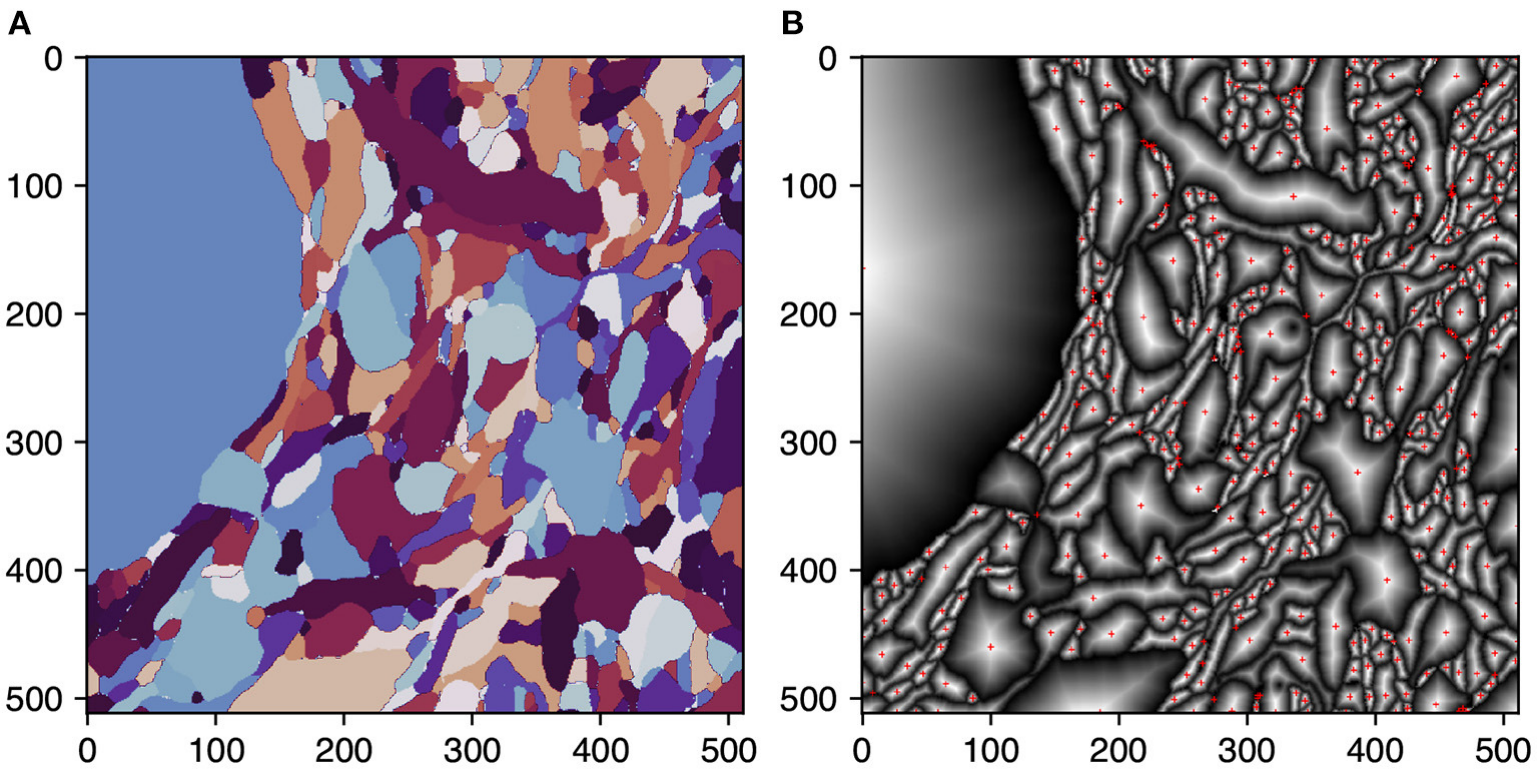
Selecting skeletonization border targets. Selection of border targets on one face of a rectangular 3D segmentation cutout. This process is applied to all six faces. Each face is overlapped by one voxel with the neighboring task to ensure perfect mating of adjacent skeleton traces. **(A)** The segmentation on the first slice of a task's 3D image. **(B)** Per segment normalized distance transform. Red crosses indicate the peak transform values and are the border targets.

Igneous ensures reliable connections between skeleton chunks by using single voxel overlap between tasks and ensuring that skeletons generated on each side of the border will meet at the same voxel. This property allows them to be trivially stitched before post-processing. This is accomplished by changing the TEASAR algorithm to first target borders before proceeding normally. A border target is defined for each 2D connected component extracted from the boundary cross-section of each shape. Each target is defined as the voxel containing the peak euclidean distance transform of each connected component (see Figure 5). This metric ensures that, unlike a centroid, the target voxel resides within the component.

To break ties between peak voxels, we use topological features to ensure that the criteria are coordinate-frame independent as the connecting coordinate frames are mirrored on each side of the task (top & bottom, left & right, back & front). The tie-breaking features in order of precedence:

1. Shape Centered: The peak value closest to the centroid of the current shape.
2. “Centerness”: Between values that are tied for shape centering, we choose the value closest to the center of the face.
3. “Cornerness”: Between values that are equidistant from both the shape centroid and the image centroid, choose the values that are closest to the corners of the image.
4. “Edgeness”: Of the values equidistant from the shape center, the image center, and corners, pick the one closest to the edge of the image.

These criteria work well for most shapes but a small number of shapes such as an annulus, X, square shape, or specially constructed irregular shapes centered at the image center will generate up to eight candidate points. We haven't implemented a final tie-breaker, but it could be resolved by introducing an asymmetric criterion (such as selecting the top-left peak). This can be made to work if it alternates between top-left and top-right depending on the orientation (e.g., up vs. down) of the face, otherwise, it will introduce a disconnection.

If a shape contacts the edge or corner of the bounding box, it will generate two and three targets respectively. The adjacent tasks will also draw skeletons to each of these target points, resulting in small loops. These loops are later resolved during post-processing during which loops are removed, small extensions are pruned, and components closer together than the nearest boundary are joined.

By using a fast and memory-efficient library, a chunked skeletonization strategy, and supporting sharded skeleton production (see Condensing Files), Igneous makes mass production of quality skeletons practical. Ensuring that Kimimaro has appropriate visual context is a somewhat more difficult problem that doesn't yet have a perfect solution as the arbitrary division of the image into a regular grid can split objects. A reasonable way to manage this problem is to ensure that the amount of visual context in the image is greater than the size of the largest objects of interest. Reducing image resolution and increasing the size of each task can both increase visual context.

Similar to meshing, we often skeletonize volumes at a near-isotropic resolution. While our highest resolution segmentation is usually 4 × 4 × 40 or 8 × 8 × 40 *nm*^3^, ordinarily skeletonization is processed at 32 × 32 × 40 *nm*^3^. At lower resolutions, such as 64 × 64 × 40 *nm*^3^, we find that skeletons become noticeably de-centered from neurites. At even lower resolutions, thin processes may become disconnected.

By default, skeletonization tasks are 512^3^ voxels, which at 32 × 32 × 40 *nm*^3^ equates to 16.4 × 16.4 × 20.5 μ*m*^3^ of physical context. The interconnection scheme described above works well for wire-like objects, but more bulbous objects such as somata, require full context to produce a reasonable skeleton. It is difficult to guarantee that large objects will have full context when crudely dividing the image, so future work will be needed to refine this aspect.

### 2.6. Contrast correction

Contrast correction *via* histogram equalization on a per-Z slice basis is supported for 8 and 16-bit images with optional right and left tail clipping. This operation is two pass as statistical information about the histogram of each slice must be known before the adjustment can be made. The first pass collects sample data from patches assigned in a regular grid at a configurable fraction of the image area (default 1%). It then writes a JSON file for each slice containing the sampled histogram. A second pass of contrast correction tasks then performs the histogram equalization on a regular grid of chunks to avoid downloading an entire slice (which may be dozens of gigabytes). Downsampling is integrated into the second pass to enable rapid visualization and save work.

### 2.7. Dataset management

Managing large datasets is a problem in its own right. Simply enumerating a billion files can become a challenge when only thousands or tens of thousands of filenames can be listed per second. Nonetheless, it is frequently advantageous to move datasets to share them with collaborators, to move the data closer to the site of computation, to use a more economical storage provider, or to re-encode them with a different compression algorithm.

Igneous provides convenient commands to transfer, re-encode, re-chunk, delete, and condense large data for images, meshes, and skeletons. Transfers and deletions are often more efficient when working with sharded volumes as fewer files need to be manipulated.

#### 2.7.1. Transfer, re-chunking, and re-encoding

Image transfers divide the image into a regular grid of tasks, each of which manages the transfer of its grid space. Except in special cases, transfers download the image region and render it into an array internally before constructing the files to write. This allows images to be downsampled as they are transferred, which saves future downsampling work and makes it easier to visualize the transfer in progress as Neuroglancer can be used even when the transfer is incomplete.

At the same time, a new chunking scheme and/or encoding can be applied. For example, a 3D dataset can be converted into 2D slices or vice versa. 3D chunking is better for visualization and certain IO patterns, while 2D slices are very useful during the alignment phase. A raw encoding, for example, can be changed to a higher compression encoding such as jpeg or compresso.

If no new encoding is applied, no downsamples are generated, and the chunk size is identical, the transfer is performed very efficiently without decompressing and recompressing each file.

Mesh and skeleton transfers are simpler. They divide up the label or shard file prefix space and assign ranges to each task for transfer. In most cases this works well, but if the stored labels are clustered under a long prefix, a custom strategy may be needed.

#### 2.7.2. Deletion

Igneous deletes image data in much the same way it performs other operations. The dataset is divided into a rectangular grid with a task assigned to each grid space. The delete tasks do not require downloading or uploading data, so each task can be much larger. By default, each task will also delete the five mip levels above it. For very large datasets, it might be necessary to “superdelete” the higher mip levels in a second or third pass.

Unsharded skeletons and meshes are handled similarly to transfers. The prefix space is divided up and a prefix is assigned to each task. Shards can be deleted using Igneous, but they are usually few enough in number that an ordinary deletion command will suffice within a few minutes. For example, 10^5^ shards deleted at a rate of 300 per second will be eliminated within 6 min.

#### 2.7.3. Sharded transfers

Using a transfer command with the --sharded flag will automatically create tasks to aggregate unsharded data into sharded data. As of this writing, unsharded to unsharded, unsharded to sharded, and sharded to sharded transfers are all supported. Only sharded to unsharded is not yet supported, though it may be useful to restore random write access to a sharded dataset. Downsamples are not automatically generated for sharded image transfers.

### 2.8. Using and installing igneous

Igneous can be installed on any system meeting the following requirements (a) runs a supported cPython version (currently 3.7+) (b) runs a recent Linux, MacOS, or Windows operating system OR can run an Ubuntu Linux based Docker container (c) all workers can access a common queue *via* either FileQueue which requires a filesystem with consistent advisory file locks OR *via* internet access to AWS SQS. Installation with Python pip is simple: pip install igneous-pipeline.

Igneous can be scripted in Python or run from the command line. A typical workflow is to first select a dataset and operation, and then enqueue a set of tasks in either FileQueue or Amazon SQS from a local workstation. Then, using the execute command, which may be used on the same workstation or a cluster, the queue is selected and executed against with one worker process per an available core. Execution continues until the queue is empty. Termination can be set to automatic in the case of FileQueue, but SQS only returns the approximate number of tasks enqueued and so requires either manual monitoring or repeated polling to verify the current job is finished. Examples of Igneous CLI commands can be seen in Listing 1 and an enumeration of the available commands is available in Table 1.

**Listing 1:**
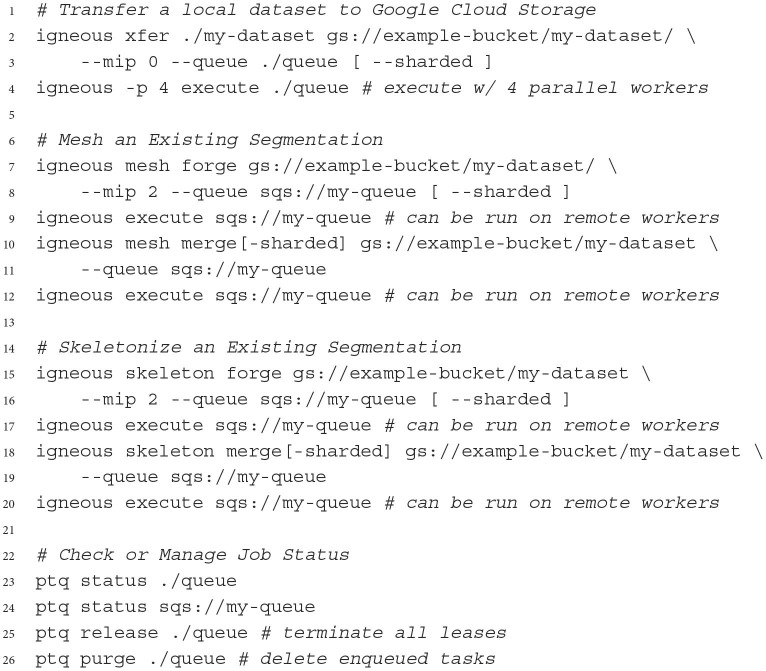
Select examples of using igneous on the command line. Square brackets indicate optional arguments.

**Table 1:** Igneous CLI commands and sub-commands.

**Command**	**Sub-commands**
design	bounds, ds-memory, ds-shape
image	contrast, downsample, rm, xfer
mesh	forge, merge, merge-sharded, rm, spatial-index, xfer
skeleton	forge, merge, merge-sharded, rm, spatial-index, xfer
execute	
view	
license	

Some commands, such as spatial-index, have deeper trees.

Parallel execution can be accomplished by any runner that can run the command igneous execute$QUEUE where $QUEUE is a path (such as ./queue or sqs://my-queue). On a local machine, parallel execution can be triggered by the -p flag which indicates the number of processes to start. For large distributed jobs, we have used Docker/Kubernetes and SLURM as runners, but many other platforms would be suitable. Queue progress can be monitored and managed *via* the co-installed ptq (short for “Python Task Queue”) command line utility.

## 3. Results

We attempted to characterize the performance of several aspects of Igneous by fully processing a segmentation of the well-known S1 dataset, by reporting the computation required by large historical runs, and reporting the efficacy of compression algorithms as applied to CREMI data (https://cremi.org/data/). We also used CREMI data to roughly characterize the quality of Igneous generated meshes and skeletons.

### 3.1. Evaluating image compression on CREMI data

As images generally comprise the bulk of storage for a given connectomics dataset, we attempted to characterize the efficacy of different compression technologies supported by Neuroglancer. We downloaded three pairs of padded test image and training segmentation datasets. We converted the HDF5 files into uncompressed Neuroglancer Precomputed volumes using CloudVolume and then re-encoded the raw volume using the igneous xfer command. For each re-encoding, we measured the computation time taken and the resultant disk space used. For decoding, we timed reading each resultant volume with CloudVolume 8.7.0 five times and reported the average.

All measurements were taken on a 2021 M1 Macbook Pro with an SSD using one process. The measurements for CREMI volumes A+, B+, and C+ were averaged into a point estimate of computation time in megavoxels per second (MVx/s) and disk space required for each encoding. All three CREMI image volumes were 8-bit 3072 × 3072 × 200 voxels (1.9 gigavoxels each, 5.7 gigavoxels total). All three CREMI segmentation volumes were all rendered as 64-bit 1250 × 1250 × 125 voxels (195 MVx each, 586 MVx total).

**Figure 6:**
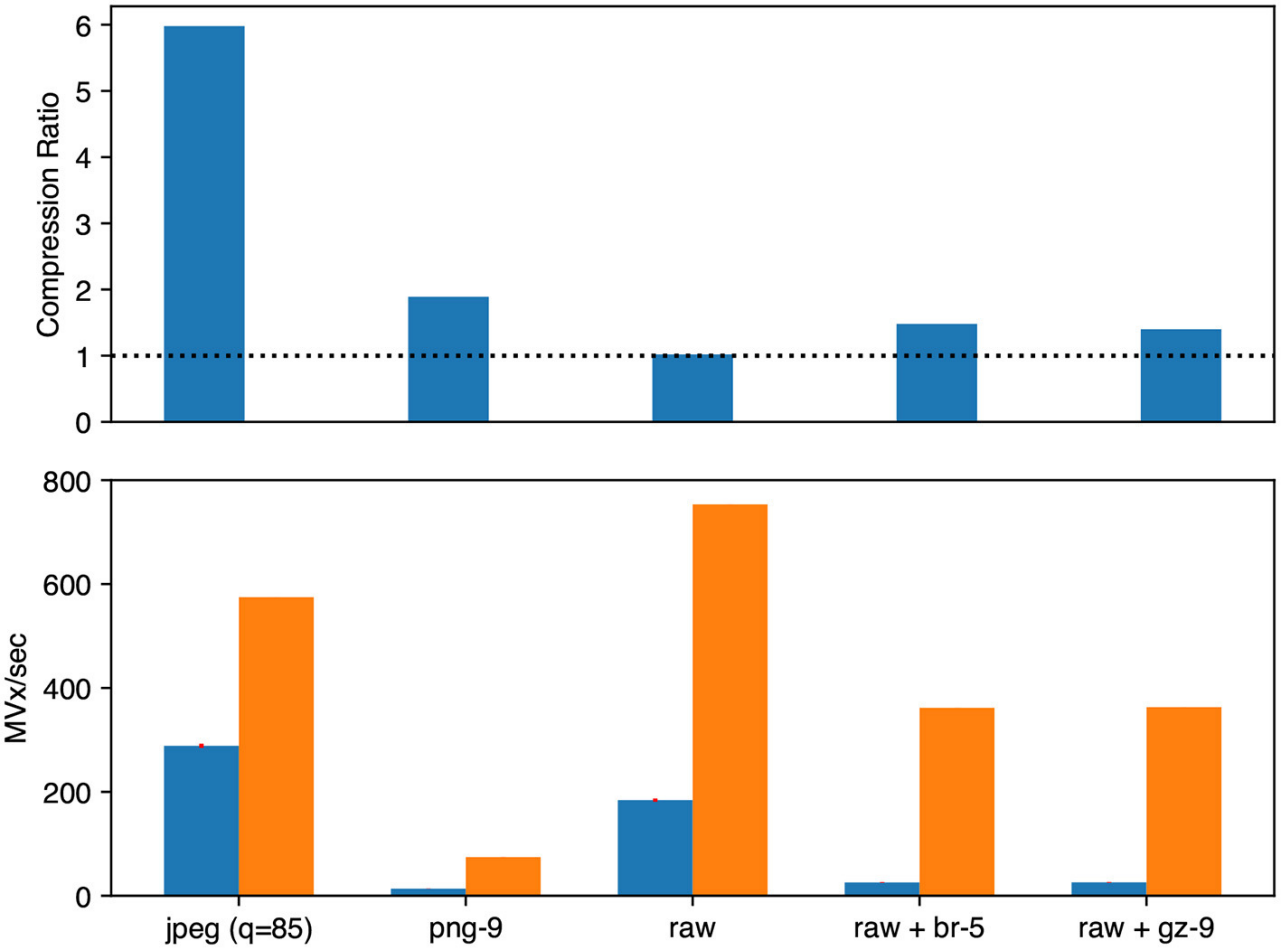
Microscopy image compression performance. We evaluated Neuroglancer compatible compression codecs against CREMI microscopy images by re-encoding an uncompressed volume with Igneous. Averages across the three images are shown. **(Top)** Compression factor (original/compressed bytes), larger is better. The dashed line indicates the level of no compression. (**Bottom**, left/blue) Encoding speed in megavoxels per second (MVx/s) (**Bottom**, right/orange). Decoding speed in MVx/s. Larger is better for all three metrics.

For microscopy images (see Figure 6), raw (uncompressed), raw+gzip, raw+brotli, jpeg, and png transfer encodings were evaluated. PNG and gzip were evaluated at compression level 9, brotli at level 5, and JPEG at 85% quality. For segmentation images, we evaluated raw (uncompressed), compresso, and compressed_segmentation encodings layered in combination with gzip level 9 and brotli level 5.

The best compression and encoding speed for images was the lossy JPEG encoding (5.98 × , 288.6 MVx/s). The best lossless compression was PNG (1.89 × ) though it was the slowest (13.7 MVx/s). raw+brotli and raw+gzip had similar encoding speeds (25.7 and 25.8 MVx/s), but raw+brotli gave a slightly smaller file size (1.40 × gzip and 1.48 × brotli).

Decoding was uniformly much faster than encoding for microscopy images. The fastest decoding time was naturally held by uncompressed data (753.4 MVx/s). jpeg was second (574.5 MVx/s). Of the lossless compression codecs, raw+gzip (363.0 MVx/s) and raw+brotli (361.8 MVx/s) were similar. png was the slowest (74.5 MVx/s).

**Figure 7:**
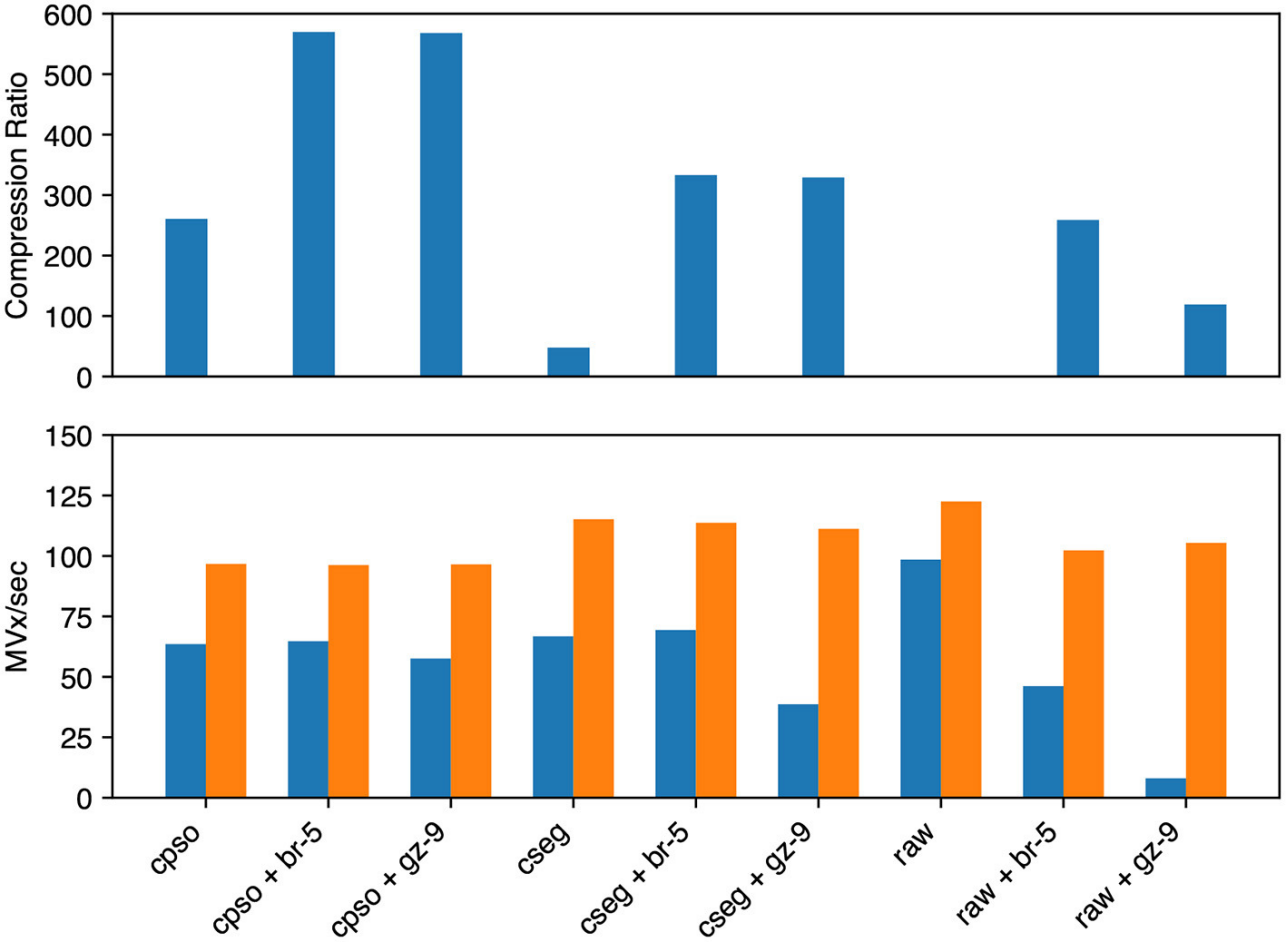
Segmentation image compression performance. We evaluated Neuroglancer compatible compression codecs against CREMI training segmentation images by re-encoding an uncompressed volume with Igneous. Averages across the three images are shown. **(Top)** Compression factor (original/compressed bytes), larger is better. (**Bottom**, left/blue) Encoding speed in megavoxels per second (MVx/s) (**Bottom**, right/orange). Decoding speed in MVx/s. Larger is better for all three metrics. Key | cpso: Compresso; cseg: compressed_segmentation; br: Brotli; gz: Gzip; raw: Uncompressed.

For segmentation (see Figure 7) we evaluated raw, compressed_segmentation, and compresso each with no compression, gzip, and brotli second stage compression. The overall best compression was given by compresso+brotli (570 × , 64.7 MVx/s). compresso+gzip gave a similar compression ratio, but was slightly slower at encoding. compressed_segmentation+brotli was slightly faster (69.4 MVx/s) but only yielded a 333 × compression ratio (58%). The fastest overall method was writing uncompressed raw arrays (98.5 MVx/s), though it is not suitable for large datasets. Using compresso+brotli resulted in an 8 × speedup and a 4.8 × compression improvement vs. the naïve raw+gzip approach.

Decoding speeds were faster than encoding speeds in all segmentation trials and showed less variability between codecs. Encoding and decoding speeds were also closer in magnitude than with microscopy image compression. compresso was slowest (96–97 MVx/s). Interestingly, it seemed to yield the same decoding speed regardless of second layer compression type (including none). raw compression was slightly faster and compressed_segmentation was the fastest encoding type (111–115 MVx/s). raw without any compression was fastest at 122.5 MVx/s.

### 3.2. Processing mouse primary somatosensory cortex (S1) segmentation

**Figure 8:**
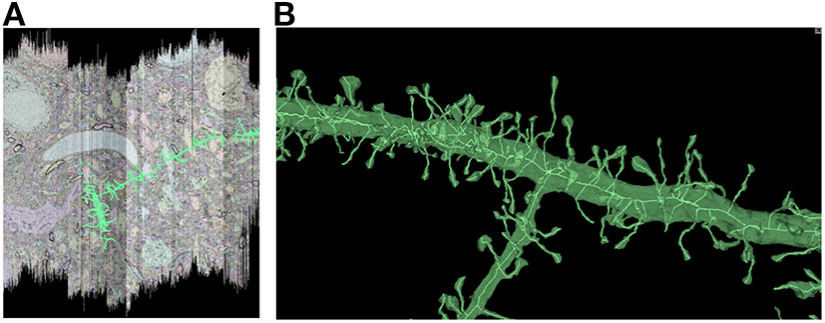
Igneous generated meshes and skeletons in S1. An example of an Igneous generated mesh and skeleton extracted from the S1 dataset displayed in Neuroglancer. **(A)** The skeleton is overlaid on a volumetric image cross section. The colors of an automatic segmentation can be faintly seen. **(B)** A close up of a semi-transparent 3D view of the same segment meshed and skeletonized. Accurate skeletonization of spines and reasonable branching behavior can be seen.

In order to characterize the computation and disk space required to process a dataset using Igneous, we processed a rough automatic segmentation of the well-known mouse S1 dataset (Kasthuri et al., 2015) which is 283.1 gigavoxels (7.4 GB gzipped) and has a resolution of 6 × 6 × 30 *nm*^3^ (see Figure 1). We downloaded the dataset from cloud storage to an 8-core 2021 M1 Macbook Pro with an SSD. The segmentation was then converted into a sharded compresso+gzip encoded image (shards do not currently support brotli) with 256 × 256 × 32 voxel chunks (Compresso is more effective on larger XY planes, hence the asymmetry). It was then unsharded downsampled to mip 5 in one step. The downsampled chunks were compresso+brotli compressed. It was then meshed in Draco compressed sharded format with a single resolution at image resolution 24 × 24 × 30 *nm*^3^, and skeletonized using parameters intended to capture spines at the same resolution. The results can be seen in Figure 8.

Table 2 shows the time and disk usage required by each stage of processing. In total, 36 core-hours were required, which works out to 7.9 gigavoxels/core-hr. The most computationally demanding tasks by far were generating mesh and skeleton fragments (“forging”) at all spatial grid points. However, in our experience, if parameters are poorly chosen or large mergers are present, skeleton merging can become demanding as well.

**Table 2:** Igneous processing time and disk usage in the S1 dataset.

**Job**	**Core-hours**	**Disk usage (GB)**
Convert segmentation	1.6	2.3 (one mip)
Downsample segmentation	1.5	5.4 (all mips)
Mesh forging (mip 2)	15.6	4.6 (fragment files)
Mesh merging	1.5	1.1 (shard files)
Skeleton forging (mip 2)	15.5	0.3 (fragment files)
Skeleton merging	0.3	0.3 (shard files)
Totals	36.0	6.8 (final files)

Quite sensibly, segmentation images including downsamples comprise the bulk of disk storage (5.4 GB), but thanks to compression, the highest resolution image (2.3 GB) isn't the single largest consumer of disk space. Instead, mesh intermediate files (4.6 GB) are the largest single item. While the intermediate fragments are simplified, they are gzip compressed while the final meshes will be merged and Draco compressed. Additionally, while meshes represent only the surface of many neighboring voxels, they do so by using three floating point numbers for the vertices plus three integers for each triangle face. For some geometries, this can inflate the size of the representation compared with the image, especially when the image is well-compressed. Skeleton fragments and final files (both 300 MB) are lightweight as expected.

6.8 GB of final compressed files remained after processing. An additional 4.6 GB of intermediate mesh fragment files and 300 MB of intermediate skeleton fragment files were also present which can be deleted. If no intermediate files are deleted, the total size of the finished dataset is 12 GB including the spatial index (which can be useful for end-users beyond the generation of meshes and skeletons).

### 3.3. Historical runs

To help illustrate Igneous's ability to scale, we provide some illustrative examples of the performance of several large jobs that have been run over the years. These jobs were run on Google Cloud Platform and Google Cloud Storage using preemptible 16-core n1-standard-16 (104 GB RAM), n1-highmem-16 (128 GB RAM), and e2-highmem-16 (128 GB RAM) machine types with one Igneous process per a core. Igneous's performance may have improved since these jobs were run.

Note that while we provide core-hours here, the total cost of these jobs depended on many factors such as the number of reads, writes, and bandwidth. Often the most expensive cost was writing unsharded meshes or skeletons as this would generate at least one or two files per a segmentation label resulting in hundreds of millions or billions of files generated. Hence, for large datasets we now recommend the sharded format.

Table 3 was compiled retrospectively from contemporaneous notes. It shows that Igneous scales to jobs requiring at least 16,000 cores and 1,000 machines simultaneously for grayscale image downsampling on a 95 teravoxel volume which was completed in a little over an hour in wall-clock time. The largest volume processed was 298 teravoxels which was a segmentation downsampling job and was completed in a little over 3 h wall-clock time.

**Table 3:** Historical Igneous run characteristics.

**Date**	**Job**	**Res. *nm*^3^**	**TVx**	**Tasks**	**Nodes**	**Cores**	**Core-hrs**
3/9/19	Downsample image	16 × 16 × 40	95	2.5M	1,000	16,000	19,200
4/13/19	Meshing (primary)	32 × 32 × 40	2.8	50k	250	4,000	12,700
4/19/19	Downsample segmentation	8 × 8 × 40	298	1.1M	200	3,200	10,400 (est.)
5/4/19	Meshing (primary)	32 × 32 × 40	74	175k	350	5,600	99,800
5/5/19	Meshing (merging)	32 × 32 × 40	74	3M	40	640	2,450
1/21/20	Sharded skeletonization (primary)	32 × 32 × 40	74	143k	35	560	91,000 (est.)
12/29/20	Meshing (primary)	32 × 32 × 40	11.7	112k	100	1,600	46,400
12/31/20	Meshing (merging)	32 × 32 × 40	11.7	300k	20	320	< 4,800 (est.)
3/24/22	Sharded skeletonization (primary)	32 × 32 × 40	0.4	4k	20	320	3,700 (est.)
3/24/22	Sharded skeletonization (merging)	32 × 32 × 40	0.4	32	64	1,024	330 (est.)

All runs are unsharded except where noted. The rows are drawn from four different unpublished versions of datasets. Three are drawn from different datasets generated in the course of the cubic millimeter project and one from FAFB. The merging step of the corresponding January 2020 skeletonization run was an iteratively developed and so is omitted. The final sharded skeletonization run had a feature called “fix avocados” enabled that slowed it down. Core-Hours annotated with “(est.)” are upper bound estimates. TVx stands for teravoxels. Core-Hours additionally annotated with “ < ” may be a significant overestimate.

The most computationally expensive job listed was the 5/4/19 unsharded primary mesh fragment generation step for an unpublished draft automatic segmentation of the cubic millimeter dataset (MICrONS Consortium et al., 2021) which took 99,800 core-hours. By comparison, the following merging step was much less intensive and took only 2,450 core-hours.

**Figure 9:**
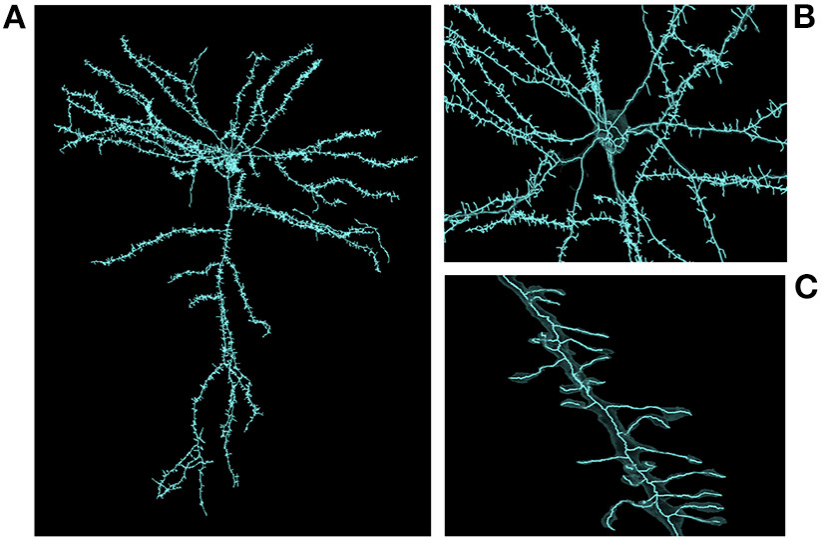
Large scale skeleton. An example skeleton extracted from an early automatic segmentation of a large subset of the cubic millimeter dataset (https://www.microns-explorer.org/cortical-mm3) displayed in Neuroglancer. This skeleton was mass produced alongside hundreds of millions more, though only a fraction of segments represent fairly complete cells. The semi-transparent silhouette of the cell's surface mesh can be seen. **(A)** A zoomed out view of the cell. **(B)** A closer view of the area around the cell body. **(C)** A close up view of one of its dendrites.

Skeletonization was similarly demanding. The most computationally expensive job listed was the 1/21/20 sharded skeleton fragment generation at an estimated 91,000 core-hours for the same dataset. The skeletonization parameters were set to capture dendritic spines. That run produced 397,770,063 skeletons in 524,288 shard files which occupied 747.6 GB of disk space. An example skeleton can be seen in Figure 9. The corresponding merging operation is not shown in Table 3 as it was run almost a year later and at the time required an iterative development cycle to increase performance over a period of months making a point estimate unhelpful.

The skeletonization run on 3/24/22 was conducted on an unpublished dataset with spine capture and “avocado fixing” enabled. This caused the primary skeletonization phase to be slower than would otherwise be expected. The merging run had the somewhat expensive “remove ticks” feature disabled, and thus preserved small extensions.

### 3.4. Characterizing mesh quality

**Figure 10:**
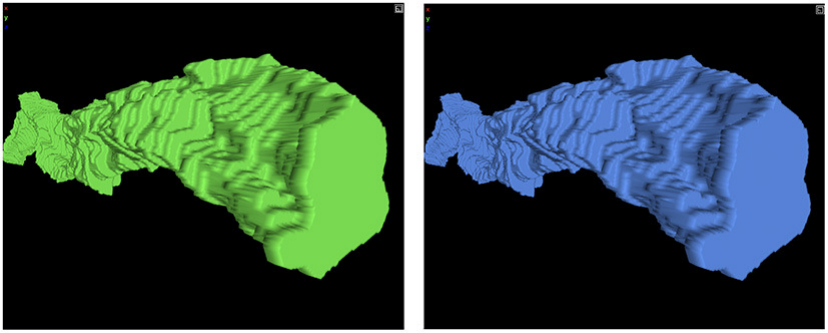
Neuroglancer screenshot of scikit-image and Igneous versions of a mesh. Unsimplified meshes produced by **(left)** scikit-image and **(right)** Igneous.

Though they are primarily for visualization, meshes may be used to derive scientific insights. Therefore, we endeavored to provide some basic characterization of mesh quality. Using the same CREMI A padded segmentation used in our image compression experiments, we compared Igneous/zmesh generated and stitched meshes to meshes generated by scikit-image^52^ using skimage.measure.marching_cubes version 0.18.1 using the lewiner algorithm. An example object that was meshed using both methods can be seen in Figure 10.

There are 37,366 unique labels and 37,828 26-connected components in the segmentation. Of these labels, 36,902 (98.76%) are smaller than 1,000 voxels while 464 (1.24%) are greater than or equal to 1,000 voxels. We generated all meshes using zmesh as both unsimplified and simplified versions from the base resolution segmentation. Simplification was performed using zmesh's built in algorithm using a triangle reduction target factor of 100 and a maximum error tolerance of 40 nanometers. scikit-image had a limitation where only objects 2 × 2 × 2 or larger could be meshed, so only 586 meshes could be generated.

We then used Trimesh^53^ version 3.10.8 to check the resultant meshes for topological integrity using the mesh.is_volume property. According to their documentation, this checks watertightness (every edge is included in two faces), having consistent winding (each shared edge going in an opposite direction), and outward facing normals. All Igneous/zmesh meshes passed this check. scikit-image meshes failed this check. It is possible that they contained degenerate triangles as they failed a convexity test. Visually, spot checks of the unsimplified zmesh and scikit-image meshes were almost indistinguishable and overlapped almost exactly in space.

In order to quantify the degree to which the zmesh meshes fairly represented the underlying segmentation, we computed the volume and centroids of all labels and plotted histograms of the ratio of mesh volume (computed with Trimesh) to voxel volume for both simplified and unsimplified meshes as seen in Supplementary Figure 1.

In this figure, it can be seen that small label volumes are often grossly underestimated by the mesh while large labels are usually underestimated within 5 or 10% of the voxel volume for unsimplified and simplified meshes respectively. Marching Cubes cuts voxel corners to create a reasonable manifold, so it is sensible that small meshes will show larger deviations while larger meshes will show smaller deviations. In the bottom right of the figure, it can be seen that many very small objects get simplified to near or actually zero volume.

In Supplementary Figure 2, we computed the difference in voxels between mesh centroids and image label centroids for zmesh unsimplified and simplified and scikit-image meshes for all labels that were valid for scikit-image. We then evaluated the labels using connected-components-3d (Silversmith, 2021) and the Trimesh centroid method (which does not rely on correct manifold topology). The maximum error for zmesh was 53.1 voxels, while for scikit-image it was 417.9 voxels. The mean error for zmesh is 4.9 with a standard deviation of 6.7 for both unsimplified and simplified meshes. For scikit-image, the mean is 8.4 and the standard deviation is 27.3. In the figure, it can be seen that all three groups are fairly similar, but scikit-image has a long tail of large errors.

### 3.5. Characterizing skeleton quality

Characterizing skeleton quality is somewhat more difficult than with meshes due to the different skeletonizations that can be proposed for a given object depending on the needs of the user. Therefore, skeletons are somewhat more subjective though there are proposed definitions for canonical skeletons based on a grass-fire analogy, the centers of maximally inscribed spheres, and other representations (Tagliasacchi et al., 2016).

We attempted to characterize Igneous produced skeletons by coarsely comparing them with other automatically traced skeletons on a well-known dataset. Unfortunately, datasets with manually traced skeletons generally do not have a per-voxel segmentation and vice-versa. Therefore, we decided to compare automatic skeletonizations of CREMI A as was done for meshes. We attempted to locate a TEASAR implementation that was able to be installed on our available hardware, that we were able to operate properly, and was not written by one of this article's authors. However, we were unable to do so. Therefore, we made our comparisons to skeletons generated by the popular binary image skeletonization procedure in Fiji (Schindelin et al., 2012), which implements the 1994 voxel thinning algorithm by Lee and Kashyap (1994).

We used Igneous to process CREMI A at 16 × 16 × 40 *nm*^3^ resolution using the parameters const 50, scale 3, soma-accept 3500, soma-detect 1100, soma-const 300, and soma-scale 1 on all segments with greater than or equal to 1,000 voxels. We did not use the short extension (“tick”) elimination feature (though it may have slightly improved some skeletons). For Fiji, we processed the segmentation using the same size threshold at the same resolution into a series of binary TIFF files and then batch processed them. The resultant thinned binary images were processed into SWC files and then converted into Neuroglancer Precomputed skeletons that were correctly offset into the same space as the Igneous skeletons. We visually confirmed *via* spot checks that both sets of skeletons appeared in Neuroglancer and seemed on-balance reasonable in their topology and location in space.

We then made several comparisons between these skeletons to characterize them. First, we used the Trimesh 3.10.8 library to check the number of points that lay outside of their enclosing (zmesh unsimplified) mesh. Nine skeletons were unable to be compared due to Trimesh repeatedly crashing during the computation. In total, 332 segments were able to be compared out of 341. 0.3% of all vertices for both Fiji and Igneous skeletons lay outside the mesh. The existence of this small quantity may be due to small differences between the 16 × 16 × 40 *nm*^3^ resolution and the meshes created at 4 × 4 × 40 *nm*^3^.

**Figure 11:**
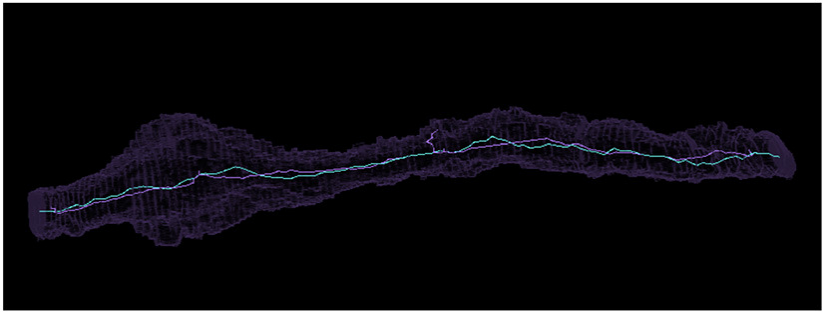
Comparing a similar voxel thinning and Igneous skeleton. Above is pictured a Neuroglancer screenshot of a meshed object in silhouette and its skeleton visualized as a bright line produced by (cyan) Igneous and (purple) voxel thinning *via* Fiji's Skeletonize3d routine.

In Supplementary Figure 3, we compared the difference in centroids, ratio of cable lengths, and difference in number of terminal points (vertices with only one connecting edge) for each set of skeletons. It can be seen that the maximum difference between centroids is 2,172 nm, though most are much less than that. The average distance between centroids is 188 nm with a standard deviation of 244 nm. We visually inspected the twelve segments more than 676 nm (two standard deviations larger) than the mean to determine their issues. In four cases, the thinning algorithm only skeletonized one of multiple connected components, leading to a much shorter cable length. In six cases, the thinning algorithm created a complex structure we referred to as a “beehive” (see Supplementary Figure 4) that added extraneous path length. Three cases were more ambiguous as to which was the better skeleton, but the thinning algorithm preserved more branches and holes. A more typical case where both thinning and Igneous created reasonable skeletons can be seen in Figure 11.

The mean cable length was 4778.7 nm (stddev 3358.5 nm) for Igneous skeletons and 6613.5 nm (stddev 5584.4 nm) for thinned skeletons. 281 (85.49%) Igneous skeletons were shorter than their thinned skeleton counterpart and 51 (15.41%) were larger. The outlier on the right hand side of the middle subplot of Supplementary Figure 3 is a near spherical object that was skeletonized across the diameter by Igneous but was reduced to almost a point by thinning.

As can be seen in the last panel, the voxel thinning skeletons had many more terminal points. A terminal point is a vertex that has only one edge connecting it to the rest of the skeleton. In total, the thinned skeletons had 3541 terminal points while the Igneous skeletons had 1169 (or a 303% difference). The average Igneous skeleton had 3.46 terminal points (stddev 9.65), while thinned skeletons had on average 10.67 points (stddev 3.71).

## 4. Discussion

Igneous is a tool for processing large 3D images using a dependency-free distributed approach that scales to at least tens of thousands of cores operating simultaneously. While many labs may produce their data sets through one-off scripts, Igneous provides a proven, clean, efficient, scalable, and documented method for contrast correction, image pyramid construction, multi-resolution meshing, skeletonization, and data management.

Igneous has been developed since 2017 and used within our lab to process a near petascale cubic millimeter of brain tissue. We have shown in practice this software processes millions of tasks and hundreds of teravoxels successfully. Our historical log shows that Igneous scales on cloud infrastructure very well, demonstrating the use of sixteen thousand cores on a thousand preemptible machines simultaneously to rapidly complete a large job. It has also been an important tool for moving and deleting copies of datasets. Large datasets often need to be moved to secure cheaper storage or to locate them closer to their next job's cluster.

To provide some confidence in the output of Igneous, we compared its outputs to popular packages. For meshes, we compared Igneous to the scikit-image package. We found that the distribution of centroids appeared similar for both unsimplified and simplified Igneous meshes compared with scikit-image. Igneous's zmesh package was able to handle small meshes that scikit-image was not able to handle. We also checked the meshes and found that they were all watertight and reasonably volumetric objects, but some scikit-image meshes failed these tests. zmesh also had a smaller maximum centroid error than scikit-image when compared with image centroids.

For skeletons, we compared Igneous to Fiji's Skeletonize3d routine, which is a voxel thinning type algorithm. It is more subjective to say one skeleton is better than another since that determination depends on what elements of a shape one is interested in. However, we can say broadly that Igneous produced simpler skeletons than Skeletonize3d and handled disconnected components. There were also fewer skeletons that appeared obviously wrong (e.g., no Igneous skeletons had a “beehive” failure mode). On the other hand, the thinning algorithm preserved certain topological features that Igneous is unable to represent, such as holes, and more frequently found small extensions. However, Igneous's settings can be adjusted to find more extensions. Igenous skeletons could have been further simplified by using the short extension elimination feature.

Igneous' output has already been used by neuroscientists. They have already made several scientific discoveries by both visualization and quantitative analysis (Wilson et al., 2019; Turner et al., 2022), with a few more uses documented in pre-prints (Schneider-Mizell et al., 2020; Buchanan et al., 2021). More papers are expected to be published in the future.

In particular, to our knowledge, no other published connectomics tool is capable of mass producing multi-resolution meshes or skeletons economically at the scale of hundreds of millions or billions of objects (some non-public tools have been shown to scale, but their economy is unknown). Accomplishing this required optimizing both in-core operations (with zmesh for meshing and kimimaro for skeletonization) and out-of-core operations. A major advantage these in-core libraries have over other implementations is that they are natively multi-label and are able to process entire segmentation cutouts in a single pass of the algorithm. To this are added out-of-core improvements with the provision of dependency-free parallelism, reliable stitching, and resolution of issues at task boundaries. As meshes and skeletons are both produced directly from segmentation images, there exists no dependency between them and they can be produced independently of each other.

It's a bit odd that quality multi-label versions of these algorithms have not previously appeared. However, this is less mysterious when it is considered that multi-label segmentation data only began appearing in bulk with the advent of the large-scale application of convolutional neural networks in the last decade. Connectomics segmentations are probably unique in both the size of the datasets and the density of labeled objects within each volume. Thus, it was previously possible to get sufficient performance out of binary versions of these algorithms.

Igneous's focus on computational efficiency is important for not only visualizing the largest connectomics projects but also for making it possible for smaller labs to perform investigations that would otherwise be out of reach. Our experiments showed that the S1 dataset's segmentation can be fully processed in only 36 core-hours on a single machine. Igneous's unique ability to use compresso for segmentation compression makes it much easier to store and transmit segmentations. Its ability to condense datasets into a much smaller number of shard files for images, meshes, and skeletons means that many different filesystems will be able to cope with big data. The techniques utilized are general and thus they can be incorporated into other systems or allow Igneous to be extended to work with other systems.

Nonetheless, there are areas that we wish to improve. Igneous does not yet support all Neuroglancer features. In particular, Igneous does not yet support annotations (though we intend to). Another problem concerns the import of raw data into Neuroglancer. As each initial dataset is often boutique in its organization and formatting, we have not yet found a standardized way to assist users in importing their raw data before Igneous can be used. However, by studying existing software, we hope to implement a method that will neatly fit into the connectomics ecosystem.

While our skeletons are of mostly high quality in thin processes, in bulbous regions such as somata, they are less organized, especially if the region was not skeletonized with full context. Parallel traces can also occur along wires that run parallel to the edges of the task grid. We hope to address these issues with future refinements.

This article also adds useful guidance for tuning both image and segmentation compression for datasets similar to CREMI. We found that for lossless compression, PNG was somewhat surprisingly better for image compression than the simple application of gzip or brotli though it was much slower for both encoding and decoding. For connectomics segmentation, we found that the overall best choice was compresso+brotli compression which to the extent of our knowledge has very low actual usage due in part to the relatively recent appearance of a practical implementation of the codec. compresso+brotli was slightly slower than compressed_segmentation+brotli at decoding but had a significantly higher compression ratio.

Igneous provides a foundational tool for reliably scaling, visualizing, analyzing, and managing connectomics datasets. It provides unique publicly accessible capabilities for large-scale meshing and skeletonization and for segmentation compression. We expect it to be an indispensable tool as connectomics datasets, such as the whole mouse brain, attain exascale.

## Data availability statement

The code for Igneous and required libraries can be found on PyPI (https://pypi.org/project/igneous-pipeline/) and GitHub (https://github.com/seung-lab/igneous) under GPL3+ licensure. A pre-built docker image can be found on DockerHub (https://hub.docker.com/repository/docker/seunglab/igneous). zmesh can be found at https://github.com/seung-lab/zmesh. Image data used for image compression, meshing, and skeletonization experiments can be found at https://cremi.org/data/. The S1 processing experiment was performed on an unpublished automatic segmentation of https://neurodata.io/data/kasthuri15/. The large scale skeletonization run was performed on an unpublished automatic segmentation of the “minnie65” subset of https://www.microns-explorer.org/cortical-mm3.

## Author contributions

WS is the primary developer of Igneous and wrote the manuscript. AZ is the author of the C++ core algorithms and Cython wrapper that were further developed by WS, NK, AZ, and JW to become zmesh. WS and JB developed the skeletonization pipeline. IT and WS initiated the project in 2017. NK contributed improvements to the meshing code. JW contributed the design for task distribution *via* a dependency-free cloud queue. HS and JW contributed to the study design. All authors contributed to the article and approved the submitted version.

## Funding

This research was supported by the Intelligence Advanced Research Projects Activity (IARPA) *via* Department of Interior/ Interior Business Center (DoI/IBC) contract number D16PC0005, NIH/NIMH (U01MH114824, U01MH117072, and RF1MH117815), NIH/NINDS (U19NS104648 and R01NS104926), NIH/NEI (R01EY027036), and ARO (W911NF-12-1-0594). The U.S. Government is authorized to reproduce and distribute reprints for Governmental purposes notwithstanding any copyright annotation thereon.

## Conflict of interest

NK is employed by Zetta AI L.L.C. HS has financial interests in Zetta AI L.L.C. This study received assistance from Google, Amazon, and Intel. These companies were not involved in the study design, collection, analysis, interpretation of data, the writing of this article, or the decision to submit it for publication. The remaining authors declare that the research was conducted in the absence of any commercial or financial relationships that could be construed as a potential conflict of interest.

## Publisher's note

All claims expressed in this article are solely those of the authors and do not necessarily represent those of their affiliated organizations, or those of the publisher, the editors and the reviewers. Any product that may be evaluated in this article, or claim that may be made by its manufacturer, is not guaranteed or endorsed by the publisher.

## Author disclaimer

The views and conclusions contained herein are those of the authors and should not be interpreted as necessarily representing the official policies or endorsements, either expressed or implied, of IARPA, DoI/IBC, or the U.S. Government.

## Footnotes

^1^ https://github.com/seung-lab/igneous

^2^ https://github.com/google/neuroglancer/tree/master/src/neuroglancer/datasource/precomputed

^3^ https://github.com/aplbrain/mesh-deco

^4^ https://github.com/hsidky/neurogen

^5^ https://github.com/janelia-cosem/multiresolution-mesh-creator

^6^ https://github.com/SridharJagannathan/pyroglancer

^7^ https://github.com/janelia-flyem/NeuTu/blob/a913db28719e328875f5017019cfcac16517cd5b/neurolabi/gui/zstackskeletonizer.cpp

^8^ https://github.com/funkey/skeletopyze/

^9^ https://github.com/seung-lab/skeletonization/

^10^ https://imagej.net/plugins/skeletonize3d

^11^ https://github.com/T4mmi/ITKThickness3D

^12^Neuroglancer also supports a few other formats but they are not precisely image file formats.

^13^ https://github.com/saalfeldlab/n5

^14^ https://nifti.nimh.nih.gov/nifti-1/

^15^ https://github.com/saalfeldlab/render

^16^ https://catmaid.readthedocs.io/en/stable/importing_data.html

^17^ https://github.com/janelia-flyem/dvid/wiki/Tile-Generation

^18^ https://github.com/jhuapl-boss/ingest-client

^19^ https://github.com/scalableminds/webknossos-libs/tree/master/wkcuber

^20^ https://github.com/seung-lab/python-task-queue

^21^ https://researchcomputing.princeton.edu/systems/della

^22^ https://www.docker.com/

^23^ https://kubernetes.io/

^24^ https://cloud.google.com/

^25^ https://github.com/seung-lab/cloud-files/

^26^https://cloud.google.com/storage/pricing, https://aws.amazon.com/s3/pricing/

^27^https://azure.microsoft.com/en-us/pricing/details/storage/blobs/#pricing. Azure also has a Premium tier with higher storage costs and reduced transaction costs.

^28^ https://github.com/google/neuroglancer/blob/056a3548abffc3c76c93c7a906f1603ce02b5fa3/src/neuroglancer/datasource/precomputed/sharded.md

^29^“Add Sharding Support” https://github.com/zarr-developers/zarr-python/issues/877.

^30^ http://libpng.org/pub/png/spec/1.2/PNG-Contents.html

^31^ https://jpeg.org/jpeg/

^32^Single Instruction Multiple Data: Instruction level parallelism.

^33^ https://github.com/dcwatson/deflate

^34^ https://github.com/ebiggers/libdeflate

^35^ https://github.com/seung-lab/pyspng-seunglab

^36^ https://github.com/randy408/libspng

^37^ https://github.com/nurpax/pyspng/

^38^ https://gitlab.com/jfolz/simplejpeg/

^39^ https://libjpeg-turbo.org/

^40^ https://github.com/google/neuroglancer/tree/f411a53f3ebc14172eb20614da2ccf6d14cd4905/src/neuroglancer/sliceview/compressed_segmentation

^41^ https://github.com/seung-lab/compressedseg

^42^ https://github.com/seung-lab/compresso

^43^ https://github.com/google/neuroglancer/blob/056a3548abffc3c76c93c7a906f1603ce02b5fa3/src/neuroglancer/datasource/precomputed/meshes.md

^44^ https://google.github.io/draco/

^45^ https://github.com/google/neuroglancer/blob/b6adee6703db3a7fc4fa35f09a9ace8318ee128b/src/neuroglancer/datasource/precomputed/skeletons.md

^46^Mip is short for the Latin phrase “multum in parvo” or “much in little”.

^47^ https://github.com/seung-lab/tinybrain/

^48^ https://github.com/seung-lab/zmesh

^49^ https://github.com/zlateski/zi_lib

^50^ https://github.com/seung-lab/DracoPy

^51^ https://github.com/Kramer84/pyfqmr-Fast-Quadric-Mesh-Reduction

^52^ https://scikit-image.org/

^53^ https://trimsh.org/trimesh.html

## Supplementary material

The Supplementary Material for this article can be found online at: https://www.frontiersin.org/articles/10.3389/fncir.2022.977700/full#supplementary-material We include three supplements to this text.

1. A three part video tutorial on getting started with Igneous.
2. A tabular non-comprehensive landscape overview of selected viewers, proofreading tools, and computational pipelines.
3. Supplementary figures that provide additional quantitative information comparing Igneous meshes and skeletons to scikit-image and Fiji's Skeletonize3d respectively.

### Video tutorial

We show how to take sample HDF5 electron microscopy images and segmentations from https://cremi.org/data/ and fully process them into multi-resolution, meshed, and skeletonized Neuroglancer Precomputed layers. This tutorial covers the simplest processing pathway and does not cover producing sharded versions of these data types, parameter selection, nor distributed processing.

The tutorial consists of three parts:

1. Creating a base Neuroglancer Precomputed image and installing Igneous.
2. Creating multi-resolution image layers with Igneous (“downsampling”).
3. Creating meshes and skeletons.

### Selected visualization, proofreading, and computational landscape overview

To better help the reader understand Igneous' relationship to other tools, we compiled a table of selected visualization, proofreading, and computational tools and evaluated them along dimensions mentioned in the Related Work section. Though it includes many entries, this table should not be considered comprehensive.

A few tools mentioned in this table were not previously mentioned in this article, so we will give their citations here: SABER^54^, SharkViewer^55^, and Paintera.^56^

For more information about the visualization landscape, please consult Beyer et al. (2013, 2022) and Berger et al. (2018) which contain additional descriptions.

### Supplementary figures

We include additional figures comparing meshes and skeleton quality to established libraries.

1. Ratio of mesh volume to voxel volume for zmesh meshes (both simplified and unsimplified).
2. Comparison of zmesh mesh centroids to label centroids.
3. Comparison of voxel thinning and Igneous skeleton properties.
4. Example of a “beehive” skeleton.

## References

[B1] Abbott L. F. Bock D. D. Callaway E. M. Denk W. Dulac C. Fairhall A. L.. (2020). The mind of a mouse. Cell 182, 1372–1376. 10.1016/j.cell.2020.08.010 32946777

[B2] Ai-Awami A. K. Beyer J. Haehn D. Kasthuri N. Lichtman J. W. Pfister H.. (2016). NeuroBlocks–visual tracking of segmentation and proofreading for large connectomics projects. IEEE Trans. Visual. Comput. Graph. 22, 738–746. 10.1109/TVCG.2015.2467441 26529725

[B3] Alakuijala J. Szabadka Z. (2016). Brotli Compressed Data Format. Internet Engineering Task Force. 10.17487/rfc.7932

[B4] Anderson J. Mohammed S. Grimm B. Jones B. Koshevoy P. Tasdizen T.. (2011). The Viking viewer for connectomics: scalable multi-user annotation and summarization of large volume data sets. J. Microsc. 241, 13–28. 10.1111/j.1365-2818.2010.03402.x 21118201 PMC3017751

[B5] Balaji S. B. Krishnan M. N. Vajha M. Ramkumar V. Sasidharan B. Kumar P. V. (2018). Erasure coding for distributed storage: an overview. Sci. China Inform. Sci. 61, 100301. 10.1007/s11432-018-9482-6

[B6] Behnel S. Bradshaw R. Citro C. Dalcin L. Seljebotn D. S. Smith K. (2011). Cython: the best of both worlds. Comput. Sci. Eng. 13, 31–39. 10.1109/MCSE.2010.118

[B7] Berg S. Kutra D. Kroeger T. Straehle C. N. Kausler B. X. Haubold C.. (2019). Ilastik: Interactive machine learning for (bio)image analysis. Nat. Methods 16, 1226–1232. 10.1038/s41592-019-0582-9 31570887

[B8] Berger D. R. Seung H. S. Lichtman J. W. (2018). VAST (volume annotation and segmentation tool): efficient manual and semi-automatic labeling of large 3D image stacks. Front. Neural Circ 12, 88. 10.3389/fncir.2018.00088 30386216 PMC6198149

[B9] Beyer J. Al-Awami A. Kasthuri N. Lichtman J. W. Pfister H. Hadwiger M. (2013). ConnectomeExplorer: query-guided visual analysis of large volumetric neuroscience data. IEEE Trans. Visual. Comput. Graph. 19, 2868–2877. 10.1109/TVCG.2013.142 24051854 PMC4296725

[B10] Beyer J. Troidl J. Boorboor S. Hadwiger M. Kaufman A. Pfister H. (2022). “A survey of visualization and analysis in high-resolution connectomics,” in Computer Graphics Forum, Vol. 41 (Rome). 10.1111/cgf.14574

[B11] Bitter I. Kaufman A. Sato M. (2001). Penalized-distance volumetric skeleton algorithm. IEEE Trans. Visual. Comput. Graph. 7, 195–206. 10.1109/2945.942688

[B12] Boergens K. M. Berning M. Bocklisch T. Bräunlein D. Drawitsch F. Frohnhofen J.. (2017). webKnossos: efficient online 3D data annotation for connectomics. Nat. Methods 14, 691–694. 10.1038/nmeth.4331 28604722

[B13] Buchanan J. Elabbady L. Collman F. Jorstad N. L. Bakken T. E. Ott C.. (2021). Oligodendrocyte precursor cells prune axons in the mouse neocortex. bioRxiv. 10.21203/rs.3.rs-581121/v1 PMC9889886 36417438

[B14] Cardona A. Saalfeld S. Schindelin J. Arganda-Carreras I. Preibisch S. Longair M.. (2012). TrakEM2 software for neural circuit reconstruction. PLoS ONE 7, e38011. 10.1371/journal.pone.0038011 22723842 PMC3378562

[B15] Dorkenwald S. McKellar C. E. Macrina T. Kemnitz N. Lee K. Lu R.. (2021). FlyWire: Online community for whole-brain connectomics. Nat. Methods 19, 1–10. 10.1101/2020.08.30.274225 34949809 PMC8903166

[B16] Dorkenwald S. Schneider-Mizell C. Collman F. (2020). Sdorkenw/MeshParty: V1.9.0. Zenodo.

[B17] Fiala J. C. (2005). *Reconstruct*: a free editor for serial section microscopy. J. Microsc. 218, 52–61. 10.1111/j.1365-2818.2005.01466.x 15817063

[B18] Garland M. Heckbert P. S. (1997). “Surface simplification using quadric error metrics,” in Proceedings of the 24th Annual Conference on Computer Graphics and Interactive Techniques - *SIGGRAPH '97* (Los Angelas, CA: ACM Press), 209–216. 10.1145/258734.25884929058486

[B19] Haehn D. Knowles-Barley S. Roberts M. Beyer J. Kasthuri N. Lichtman J. W.. (2014). Design and evaluation of interactive proofreading tools for connectomics. IEEE Trans. Visual. Comput. Graph. 20(12):2466–2475. 10.1109/TVCG.2014.2346371 26356960

[B20] Helmstaedter M. Briggman K. L. Denk W. (2011). High-accuracy neurite reconstruction for high-throughput neuroanatomy. Nat. Neurosci. 14, 1081–1088. 10.1038/nn.2868 21743472

[B21] Helmstaedter M. Briggman K. L. Turaga S. C. Jain V. Seung H. S. Denk W. (2013). Connectomic reconstruction of the inner plexiform layer in the mouse retina. Nature 500, 168–174. 10.1038/nature12346 23925239

[B22] Hider R. Kleissas D. M. Pryor D. Gion T. Rodriguez L. Matelsky J.. (2019). The block object storage service (bossDB): a cloud-native approach for petascale neuroscience discovery. bioRxiv. 10.3389/fninf.2022.828787 PMC8885591 35242021

[B23] Hoppe H. (1999). “New quadric metric for simplifying meshes with appearance attributes,” in Proceedings Visualization '99 (San Francisco, CA: IEEE), 59–510. 10.1109/VISUAL.1999.809869

[B24] Jeong W. K. Beyer J. Hadwiger M. Blue R. Law C. Vazquez-Reina A.. (2010). Ssecrett and NeuroTrace: interactive visualization and analysis tools for large-scale neuroscience data sets. IEEE Comput. Graph. Appl. 30, 58–70. 10.1109/MCG.2010.56 20650718 PMC2909612

[B25] Kasthuri N. Hayworth K. J. Berger D. R. Schalek R. L. Conchello J. A. Knowles-Barley S.. (2015). Saturated reconstruction of a volume of neocortex. Cell 162, 648–661. 10.1016/j.cell.2015.06.054 26232230

[B26] Katz W. T. Plaza S. M. (2019). DVID: distributed versioned image-oriented dataservice. Front. Neural Circ. 13, 5. 10.3389/fncir.2019.00005 30804760 PMC6371063

[B27] Kim J. S. Greene M. J. Zlateski A. Lee K. Richardson M. Turaga S. C.. (2014). Space-time wiring specificity supports direction selectivity in the retina. Nature 509, 331–336. 10.1038/nature13240 24805243 PMC4074887

[B28] Kremer J. R. Mastronarde D. N. McIntosh J. R. (1996). Computer visualization of three-dimensional image data using IMOD. J. Struct. Biol. 116, 71–76. 10.1006/jsbi.1996.0013 8742726

[B29] Lee T.-C. Kashyap R. L. (1994). “Building skeleton models via 3-D medial surface/axis thinning algorithms,” in CVGIP: Graphical Models and Image Processing (Orlando, FL: Academic Press, Inc.), 462–478. 10.1006/cgip.1994.1042

[B30] Lorensen W. E. Cline H. E. (1987). Marching cubes: a high-resolution 3D surface construction algorithm. ACM SIGGRAPH Comput. Graph. 21, 7. 10.1145/37402.37422

[B31] Maitin-Shepard J. Baden A. Silversmith W. Perlman E. Collman F. Blakely T.. (2021). Google/Neuroglancer. Zenodo.

[B32] Matejek B. Haehn D. Lekschas F. Mitzenmacher M. Pfister H. (2017). “Compresso: efficient compression of segmentation data for connectomics,” in Medical Image Computing and Computer Assisted Intervention – *MICCAI 2017*, eds M. Descoteaux, L. Maier-Hein, A. Franz, P. Jannin, D. L. Collins, and S. Duchesne (Cham: Springer International Publishing), 781–788. 10.1007/978-3-319-66182-7_89

[B33] Matejek B. Wei D. Wang X. Zhao J. Palágyi K. Pfister H. (2019). “Synapse-aware skeleton generation for neural circuits,” in Medical Image Computing and Computer Assisted Intervention?MICCAI 2019, eds D. Shen, T. Liu, T. M. Peters, L. H. Staib, C. Essert, S. Zhou, P. T. Yap, and A. Khan (Cham: Springer International Publishing), 227–235. 10.1007/978-3-030-32239-7_26

[B34] MICrONS Consortium Bae, J. A. Baptiste M. Bodor A. L. Brittain D. Buchanan J.. (2021). Functional connectomics spanning multiple areas of mouse visual cortex. bioRxiv. 10.1101/2021.07.28.454025 PMC11981939 40205214

[B35] Miles A. Bussonnier M. Moore J. Fulton A. Bourbeau J. Onalan T.. (2022). Zarr-Developers/Zarr-Python: None. Zenodo.

[B36] Peng H. Ruan Z. Long F. Simpson J. H. Myers E. W. (2010). V3D enables real-time 3D visualization and quantitative analysis of large-scale biological image data sets. Nat. Biotechnol. 28, 348–353. 10.1038/nbt.1612 20231818 PMC2857929

[B37] Pfister H. Kaynig V. Botha C. P. Bruckner S. Dercksen V. J. Hege H.-C.. (2014). “Visualization in connectomics,” in Scientific Visualization: Uncertainty, Multifield, Biomedical, and Scalable Visualization, eds C. D. Hansen, M. Chen, C. R. Johnson, A. E. Kaufman, and H. Hagen (London: Springer), 221–245. 10.1007/978-1-4471-6497-5_21

[B38] Pietzsch T. Saalfeld S. Preibisch S. Tomancak P. (2015). BigDataViewer: visualization and processing for large image data sets. Nat. Methods 12, 481–483. 10.1038/nmeth.3392 26020499

[B39] Reilly E. P. Garretson J. S. Gray Roncal W. R. Kleissas D. M. Wester B. A. Chevillet M. A.. (2018). Neural reconstruction integrity: a metric for assessing the connectivity accuracy of reconstructed neural networks. Front. Neuroinform. 12, 74. 10.3389/fninf.2018.00074 30455638 PMC6231021

[B40] Rose Li and Associates Inc. (2021). Brain Connectivity Workshop Series Report. Technical report, USDOE Office of Science (SC) (United States).

[B41] Saalfeld S. Cardona A. Hartenstein V. Tomančák P. (2009). CATMAID: collaborative annotation toolkit for massive amounts of image data. Bioinformatics 25, 1984–1986. 10.1093/bioinformatics/btp266 19376822 PMC2712332

[B42] Sato M. Bitter I. Bender M. Kaufman A. Nakajima M. (2000). “TEASAR: tree-structure extraction algorithm for accurate and robust skeletons,” in Proceedings the Eighth Pacific Conference on Computer Graphics and Applications (Hong Kong: IEEE), 281–449. 10.1109/PCCGA.2000.883951

[B43] Schindelin J. Arganda-Carreras I. Frise E. Kaynig V. Longair M. Pietzsch T.. (2012). Fiji: an open-source platform for biological-image analysis. Nat. Methods 9, 676–682. 10.1038/nmeth.2019 22743772 PMC3855844

[B44] Schlegel P. Kazimiers T. (2021). Schlegelp/Skeletor: Version 1.1.0. Zenodo.

[B45] Schneider-Mizell C. M. Bodor A. L. Collman F. Brittain D. Bleckert A. A. Dorkenwald S.. (2020). Chandelier cell anatomy and function reveal a variably distributed but common signal. bioRxiv. 10.1101/2020.03.31.018952

[B46] Shapson-Coe A. Januszewski M. Berger D. R. Pope A. Wu Y. Blakely T.. (2021). A connectomic study of a petascale fragment of human cerebral cortex. bioRxiv. 10.1101/2021.05.29.446289

[B47] Shearer R. W. (2009). Omni: visualizing and editing large-scale volume segmentations of neuronal tissue (thesis). Massachusetts Institute of Technology, Cambridge, MA, United States.

[B48] Silversmith W. (2021). Seung-Lab/Connected-Components-3D: Zenodo Release v1. Zenodo.

[B49] Silversmith W. Bae J. A. Li P. H. Wilson A. M. (2021a). Seung-Lab/Kimimaro: Zenodo Release v1. Zenodo.

[B50] Silversmith W. Collman F. Kemnitz N. Wu J. Castro M. Falk B.. (2021b). Seung-Lab/Cloud-Volume: Zenodo Release v1. Zenodo.

[B51] Sofroniew N. Lambert T. Evans K. Nunez-Iglesias J. Bokota G. Winston P.. (2022). Napari: A Multi-Dimensional Image Viewer for Python. Zenodo.

[B52] Tagliasacchi A. Delame T. Spagnuolo M. Amenta N. Telea A. (2016). 3D skeletons: a state-of-the-art report. Comput. Graph. Forum 35, 573–597. 10.1111/cgf.12865

[B53] Turner N. L. Macrina T. Bae J. A. Yang R. Wilson A. M. Schneider-Mizell C.. (2022). Reconstruction of neocortex: organelles, compartments, cells, circuits, and activity. Cell. 185, 1082–1100.e24. 10.1016/j.cell.2022.01.023 35216674 PMC9337909

[B54] Wanner A. A. Genoud C. Masudi T. Siksou L. Friedrich R. W. (2016). Dense EM-based reconstruction of the interglomerular projectome in the zebrafish olfactory bulb. Nat. Neurosci. 19, 816–825. 10.1038/nn.4290 27089019

[B55] Wilson A. M. Schalek R. Suissa-Peleg A. Jones T. R. Knowles-Barley S. Pfister H.. (2019). Developmental rewiring between cerebellar climbing fibers and Purkinje cells begins with positive feedback synapse addition. Cell Rep. 29, 2849–2861. 10.1016/j.celrep.2019.10.081 31775050 PMC6914268

[B56] Wu J. Silversmith W. M. Lee K. Seung H. S. (2021). Chunkflow: hybrid cloud processing of large 3D images by convolutional nets. Nat. Methods 18, 328–330. 10.1038/s41592-021-01088-5 33750934

[B57] Wu J. Turner N. Bae J. A. Vishwanathan A. Seung H. S. (2022). RealNeuralNetworks.jl: an integrated julia package for skeletonization, morphological analysis, and synaptic connectivity analysis of terabyte-scale 3D neural segmentations. Front. Neuroinform. 16, 828169. 10.3389/fninf.2022.828169 35311003 PMC8924549

[B58] Xu C. S. Januszewski M. Lu Z. Takemura S.-Y. Hayworth K. J. Huang G.. (2020). A connectome of the adult *Drosophila* central brain. Elife 9:e57443. 10.7554/eLife.57443 32880371 PMC7546738

[B59] Yoo A. B. Jette M. A. Grondona M. (2003). “SLURM: simple linux utility for resource management,” in Job Scheduling Strategies for Parallel Processing, eds D. Feitelson, L. Rudolph, and U. Schwiegelshohn (Berlin; Heidelberg: Springer), 44–60. 10.1007/10968987_3

[B60] Yushkevich P. A. Piven J. Hazlett H. C. Smith R. G. Ho S. Gee J. C.. (2006). User-guided 3D active contour segmentation of anatomical structures: significantly improved efficiency and reliability. Neuroimage 31, 1116–1128. 10.1016/j.neuroimage.2006.01.015 16545965

[B61] Zhao T. Olbris D. J. Yu Y. Plaza S. M. (2018). NeuTu: software for collaborative, large-scale, segmentation-based connectome reconstruction. Front. Neural Circ. 12, 101. 10.3389/fncir.2018.00101 30483068 PMC6243011

[B62] Zhao T. Plaza S. M. (2014). Automatic neuron type identification by neurite localization in the Drosophila medulla. arXiv preprint arXiv:1409.1892.

[B63] Zheng Z. Lauritzen J. S. Perlman E. Robinson C. G. Nichols M. Milkie D.. (2018). A complete electron microscopy volume of the brain of adult Drosophila melanogaster. Cell 174, 730–743. 10.1016/j.cell.2018.06.019 30033368 PMC6063995

