# Supplementary material for: Igneous: Distributed dense 3D segmentation meshing, neuron skeletonization, and hierarchical downsampling
Source: Front Neural Circuits. 2022 Nov 25;16:977700. doi: 10.3389/fncir.2022.977700 (PMC9732676; doi:10.3389/fncir.2022.977700)
Supplement: Supplementary file 5 [file Data_Sheet_1.docx]

Supplementary Material

# Supplementary Figures

We include some additional figures to help characterize the accuracy of our meshes and skeletons.
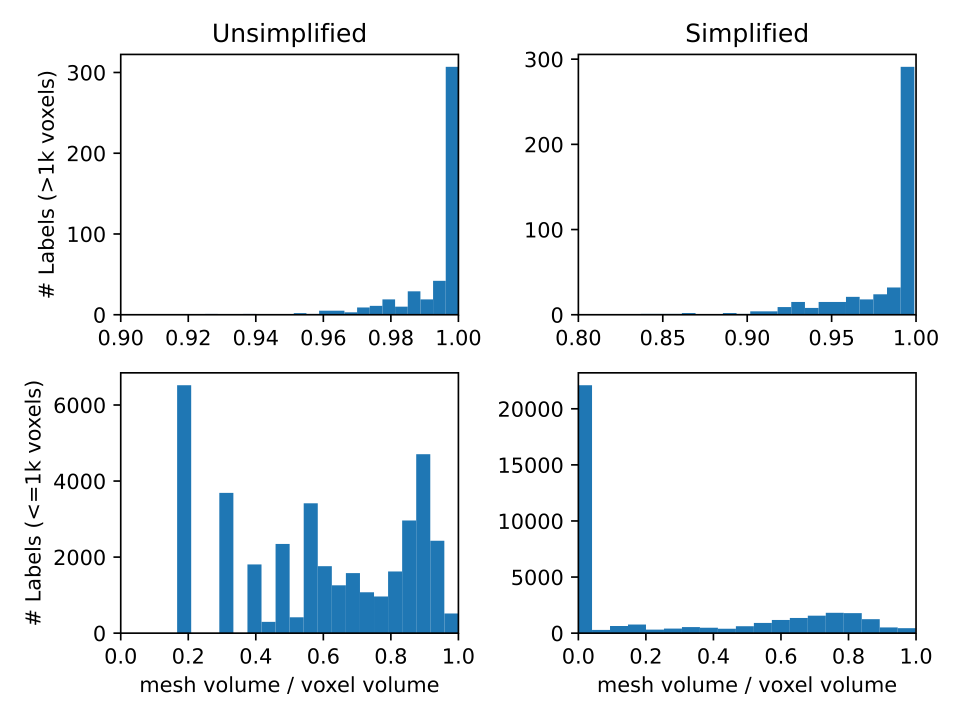


## Supplementary Mesh Figures

**Supplementary Figure 1.** The ratio of mesh volume to voxel volume for zmesh (left column) unsimplified and (right column) simplified meshes. The meshes are divided between (top row) labels greater than or equal to 1000 voxels and (bottom row) labels with fewer than 1000 voxels.


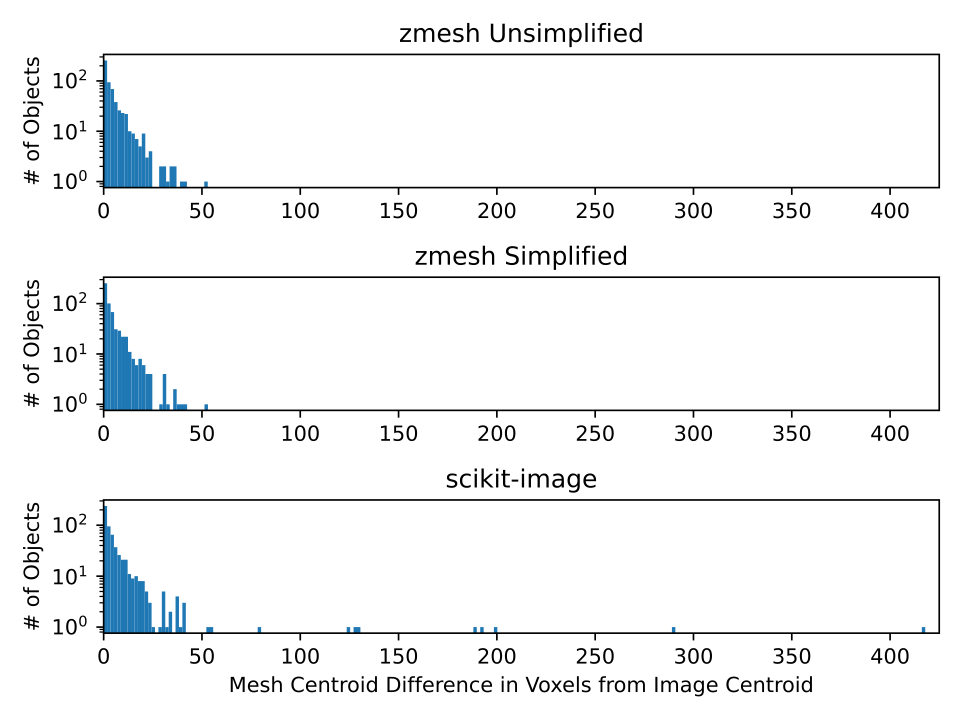


**Supplementary Figure 2.** Comparison of zmesh Mesh Centroids to Label Centroids. Euclidean distance in voxels between image derived and voxel derived centroids for (top) zmesh unsimplified meshes (middle) zmesh simplified meshes and (bottom) scikit-image unsimplified meshes.

## Supplementary Skeleton Figures

**Supplementary Figure 3.** Comparison of Voxel Thinning and Igneous Skeleton Properties We compared three important quantities between skeletons produced by Fiji’s Skeletonize3d and Ign
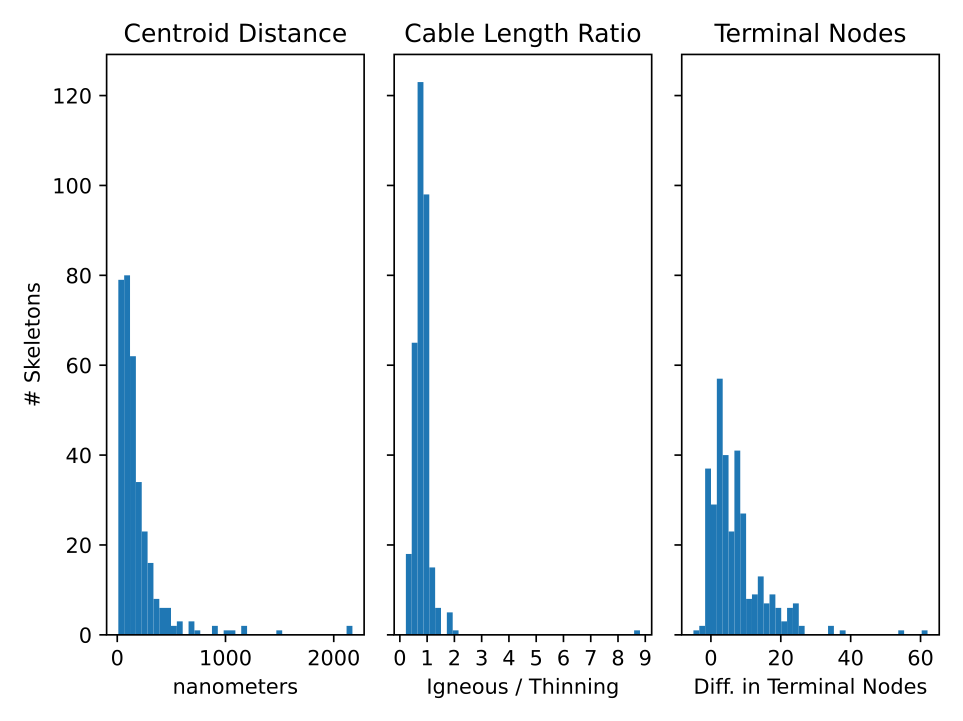
eous. (left) Difference in centroids (middle) Ratio of cable length with Igneous skeletons in the numerator (right) difference in terminal points between Skeletonize3d (the minuend) and Igneous (the subtrahend).


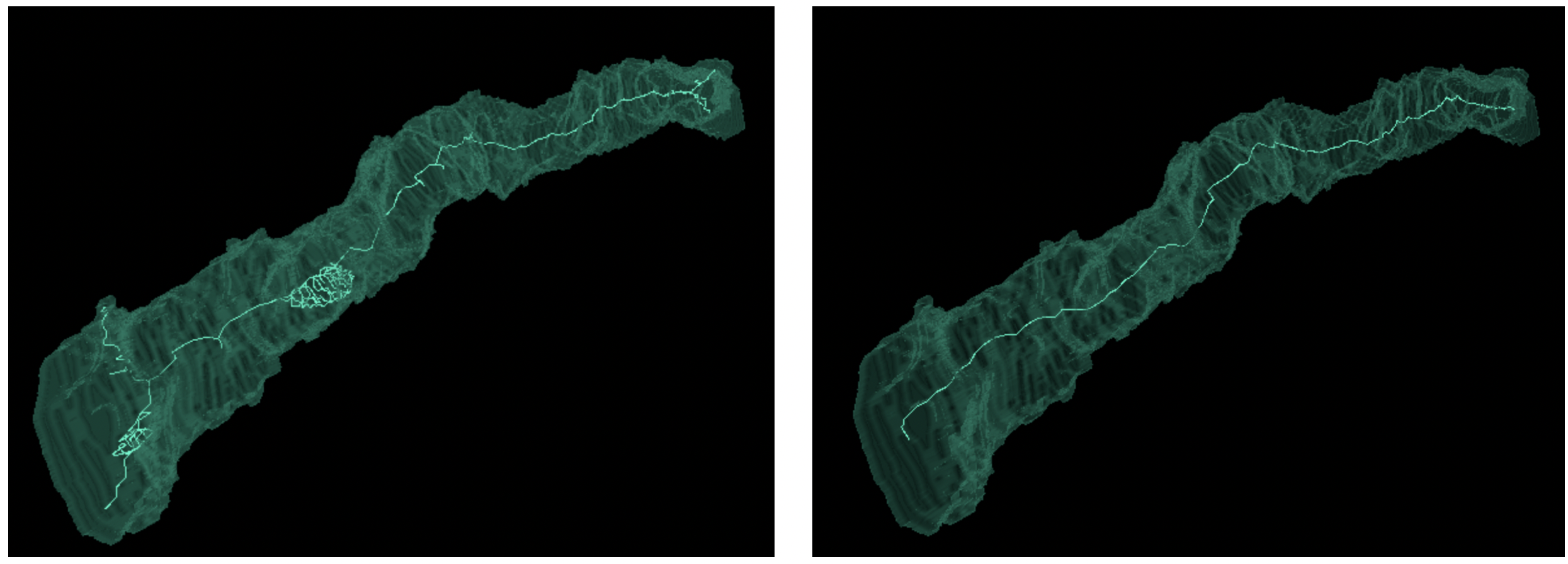
**Supplementary Figure 4.** Example of a ”Beehive” Skeleton. A Neuroglancer screenshot of (left) a skeleton produced by Fiji’s Skeletonize3d routine which includes a complex structure resembling a beehive (right) the skeleton produced by Igneous for the same object. Multiple datasets can be visualized in Neuroglancer simultaneously.
